# Mechanochemical Synthesis and Structure of Lithium Tetrahaloaluminates, LiAlX_4_ (X = Cl, Br, I): A Family of Li-Ion Conducting Ternary Halides

**DOI:** 10.1021/acsmaterialslett.1c00055

**Published:** 2021-04-20

**Authors:** Nicolás Flores-González, Nicolò Minafra, Georg Dewald, Hazel Reardon, Ronald I. Smith, Stefan Adams, Wolfgang G. Zeier, Duncan H. Gregory

**Affiliations:** 1 School of Chemistry, University of Glasgow, Joseph Black Building, Glasgow G12 8QQ, U.K.; 2 Institute for Inorganic and Analytical Chemistry, University of Münster, Correnstrasse 39, 48149 Münster, Germany; 3 Institute of Physical Chemistry, Justus-Liebig-University Giessen, Heinrich-Buff-Ring 17, D-35392 Giessen, Germany; 4 ISIS Pulsed Neutron and Muon Source, STFC Rutherford Appleton Laboratory, Didcot, Oxfordshire OX11 0QX, U.K.; 5 Department of Materials Science and Engineering, National University of Singapore, 9 Engineering Drive 1, 117575, Singapore

## Abstract

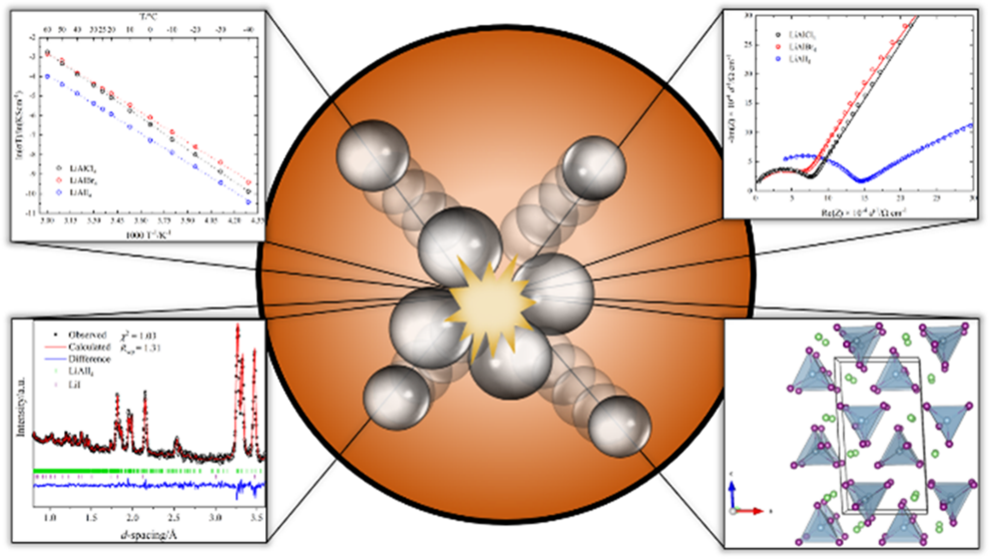

State-of-the-art oxides and sulfides with high Li-ion conductivity and good electrochemical stability are among the most promising candidates for solid-state electrolytes in secondary batteries. Yet emerging halides offer promising alternatives because of their intrinsic low Li^+^ migration energy barriers, high electrochemical oxidative stability, and beneficial mechanical properties. Mechanochemical synthesis has enabled the characterization of LiAlX_4_ compounds to be extended and the iodide, LiAlI_4_, to be synthesized for the first time (monoclinic *P*2_1_/*c*, *Z* = 4; *a* = 8.0846(1) Å; *b* = 7.4369(1) Å; *c* = 14.8890(2) Å; β = 93.0457(8)°). Of the tetrahaloaluminates, LiAlBr_4_ exhibited the highest ionic conductivity at room temperature (0.033 mS cm^–1^), while LiAlCl_4_ showed a conductivity of 0.17 mS cm^–1^ at 333 K, coupled with the highest thermal and oxidative stability. Modeling of the diffusion pathways suggests that the Li-ion transport mechanism in each tetrahaloaluminate is closely related and mediated by both halide polarizability and concerted complex anion motions.

Replacing flammable liquid electrolytes in conventional Li-ion batteries (LIBs) with solid-state alternatives could lead to a breakthrough in battery safety and longevity. Moreover, otherwise inaccessible high energy density cells (using Li-metal anodes and high-voltage cathodes) could become a reality by employing thermodynamically stable solid-state electrolytes (SSEs) in all-solid-state batteries (SSBs).^1^ Inorganic SSEs with sufficiently high ionic conductivity and chemical/electrochemical stability are almost within reach. Among them, oxide- and sulfide-based materials have been the main focus of research because of the remarkable ionic conductivity that can be achieved at room temperature. However, oxides lack mechanical strength and require high processing temperatures, whereas both the narrow electrochemical windows and limited stability of sulfides have proved challenging to their adoption as SSEs.^2,3^ Among alternatives, the complex halides, Li_3_M^III^X_6_ (M^III^ = Sc, Y, In, La, Ho, Er; X = halogen) and Li_3–*x*_M_1–*x*_Zr_*x*_Cl_6_ (M^III^ = Y, Er), are raising interest with appreciable ionic conductivity at room temperature, low activation energies for Li^+^ migration and wide electrochemical windows.^4−11^ As originally discovered in the 1990s, the synthesis of Li_3_M^III^X_6_ requires several steps including high temperature annealing.^12^ By comparison, ternary lithium-light element halides, including LiAlCl_4_, have become well-known over the past four decades on account of remarkable ionic conductivity in the solution and molten states. With liquid LiAlCl_4_·6SO_4_ showing Li^+^ ionic conductivity of >0.10 S cm^–1^ at room temperature,^13^ such halides have attracted renewed scrutiny recently,^14,15^ while in solution, LiAlX_4_ (X = Cl, Br) can surpass the Li-ion conductivity of the ubiquitous electrolyte, LiPF_6_.^16^ By contrast, the structure and conductivity of lithium tetrahaloaluminates in the solid state have scarcely been studied and only very recently has solid LiAlCl_4_ been identified as one of several promising halides for electrolytes in SSBs.^17^ In fact, the ionic conductivity of monoclinic LiAlCl_4_ was first reported by Weppner and Huggins using DC polarization measurements in 1977.^18^ At 298 K, single crystals of LiAlCl_4_ were reported with a conductivity of 1.2 × 10^–6^ S cm^–1^, increasing to 1.4 × 10^–4^ S cm^–1^ at 413 K (just below the melting temperature). The feasibility of using LiAlCl_4_ as a SSE was first demonstrated 15 years later by deploying it in a Li_*x*_TiS_2(s)_/LiAlCl_4(s)_/Li_1–*x*_CoO_2(s)_ solid-state cell (0 < *x* < 0.45) at 373 K.^19^ The cell exhibited an open-circuit potential of 2.1 V in the charged state. Moreover, it showed excellent discharge characteristics at current densities up to 0.1 mA cm^–2^ with minimal capacity loss over 100 charge–discharge cycles. Despite this promising result, there has been a lack of studies since. The corresponding bromide is uncharacterized, while the iodide has never been isolated. Consequently, the conductivity of either is unknown in the solid state.

Inspired by the recent performance of halides in the solid state and by the historically promising properties of halide salts, we were motivated to investigate the structures, stabilities and electrochemical properties of the solid lithium tetrahaloaluminates, LiAlX_4_, (X = Cl, Br, I) systematically. Here, we demonstrate how mechanochemistry can be employed to synthesize the tetrahaloaluminates in one step, without heating. This is critical for synthesizing powders of the low melting point iodide, which we could not isolate thermally. The high purity, bulk samples so-obtained have enabled us to determine the structures of the halides and to make evaluations of their Li^+^ conductivity. Both thermal and electrochemical oxidative stability have also been determined. Some preliminary hypotheses for cation conductivity mechanisms in the lithium tetrahaloaluminates can be proposed on the basis of the data.

High-purity LiAlX_4_ (X = Cl, Br, I) powders were synthesized by milling the respective component binary halides in an inert atmosphere (Tables S1 and S2 and Figures S1–S8). Although lab-based powder X-ray diffraction (PXD) provided basic crystal structure models of the halides (Figures S9–11), we undertook synchrotron PXD (SPXD) and time-of-flight (ToF) powder neutron diffraction (PND) experiments to locate the Li positions accurately and to determine anisotropic thermal displacement parameters, allowing full characterization of the underlying structural chemistry of the LiAlX_4_ materials. Figure 1 shows the profile fits for LiAlI_4_, from structure refinement against PND and SPXD data, respectively. The respective plots for the chloride and bromide analogues can be found in the Supporting Information (Figures S12, S13, and S15).

**Figure 1:**
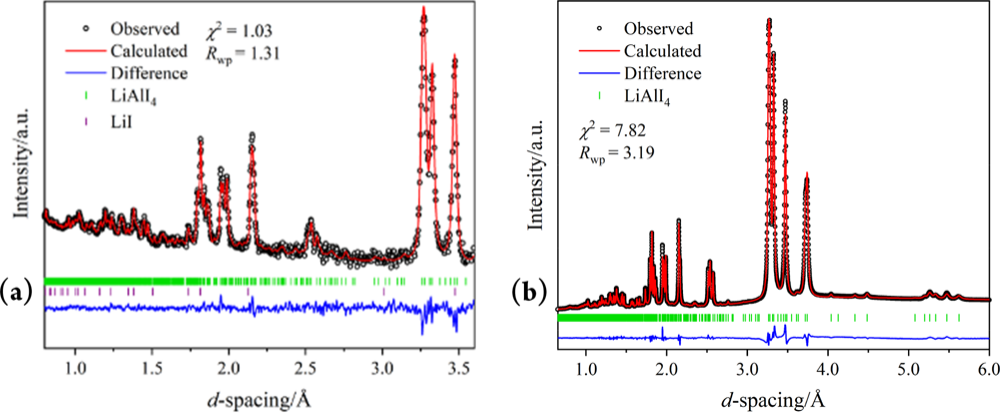
Room-temperature profile fits from Rietveld refinement of the structure of LiAlI_4_ against: (a) ToF PND data (⟨2θ = 92.59°⟩ detector bank; Polaris, ISIS),^22,23^ and (b) SPXD data (λ = 0.56466 Å; X04SA, PSI). Experimental (black), calculated (red), and difference profiles (blue) are shown; vertical markers indicate Bragg reflection positions for LiAlI_4_ (green) and LiI (purple), respectively.

Crystallographic data from these refinements are collated in Tables S3–S18. Our room temperature diffraction data confirmed that LiAlBr_4_ and LiAlI_4_ are isostructural to the chloride analogue (monoclinic, space group *P*2_1_/*c*). The structural models for the bromide and iodide were further assessed by means of the Global Instability Index (GII),^20,21^ which corroborated their plausibility with low values of 0.10 and 0.07, respectively. The crystal structure of the haloaluminates can be described as a slightly distorted *hcp* X^–^ sublattice within which Li^+^ and Al^3+^ occupy octahedral and tetrahedral interstices, respectively. The extended structure can be considered to be constructed from distorted LiX_6_ octahedra and AlX_4_ tetrahedra. Two LiX_6_ octahedra link across a common edge to form “Li_2_X_10_ dimers”. Each Li–X dimer is connected to four others by 2 axial and 2 equatorial vertices in a “trans” confirmation that creates stepped or buckled layers that propagate in all three dimensions. Meanwhile, each AlX_4_ tetrahedron is connected to one Li–X dimer via two edges and two other dimers by one vertex each (Figure 2a and b). Alternatively, considering only complex [AlX_4_]^−^ anions and Li^+^ cations, then the latter can be seen to occupy space within “pseudo-layers” between the isolated haloaluminate tetrahedra (Figure 2c).

**Figure 2:**
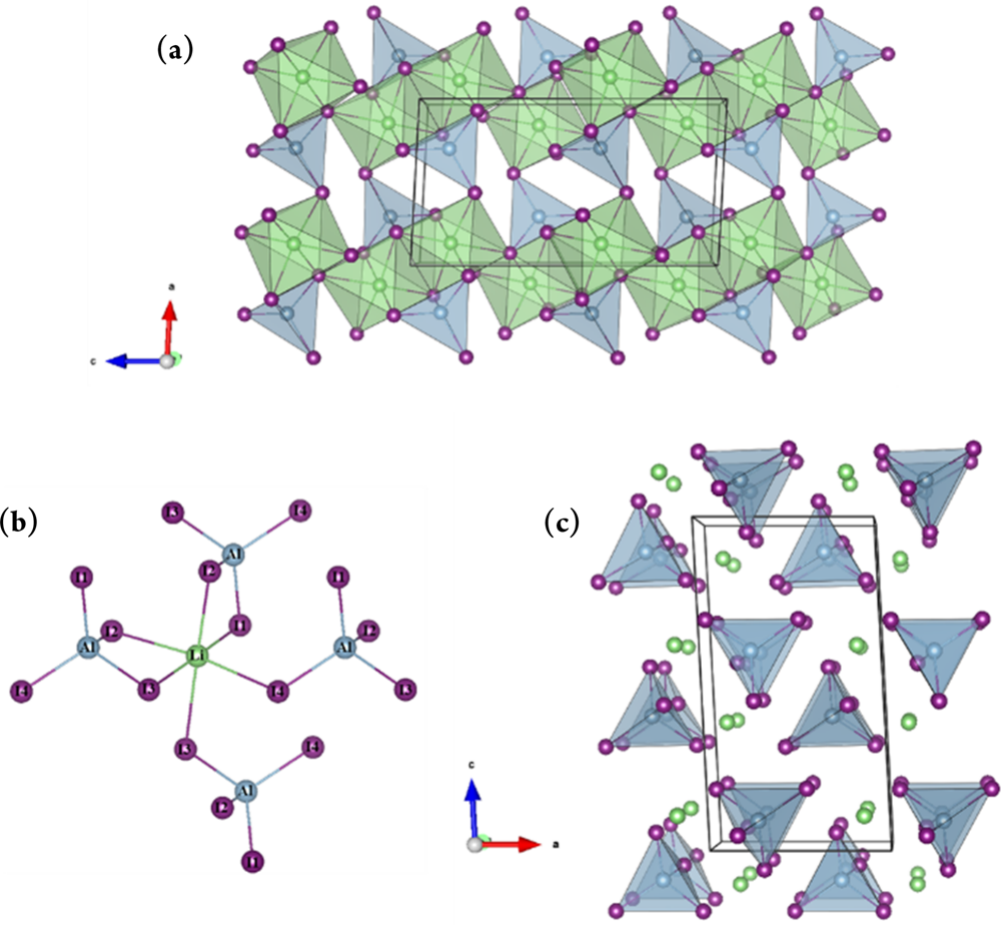
Crystal structure of mechanochemically synthesized LiAlI_4_ (*P*2_1_/*c*) projected along the *b*-axis as visualized with VESTA^26^ (a) showing a polyhedral representation of the extended structure and the linking of Li–X “dimers”, (b) showing the linkage between an LiX_6_ octahedron and neighboring AlX_4_ tetrahedra, and (c) highlighting the positions of the Li^+^ cations with respect to the isolated haloaluminate anions: Li (4*e*, green spheres), Al (4*e*, light blue spheres), and I (4*e*, purple).

The unit cell expands linearly in all three dimensions as Cl^–^ (167 pm) is replaced by Br^–^ (182 pm) and I^–^ (206 pm),^24^ and there is a concomitant increase in both the average Li–X and Al–X bond lengths. The monoclinic distortion of the cell decreases very slightly with increasing halide radius (Figure S16). Other than these expected differences in cell volume, our refinements hinted at differences between the structural models of the mechanochemically synthesized lithium tetrahaloaluminates and the previously reported structures of (thermally synthesized) LiAlCl_4_ and LiAlBr_4_ (although the latter structure was previously determined only from single crystal data at 100 K).^16^ First, attempts were made to refine the occupancy of the Li site in each halide, and although for X = Br, this did not vary from 100%, for X = Cl and I, respectively, values of 91(4)% and 94(2)% were obtained with slight reductions in *R*-factors. More interestingly, if the Li occupancy of the normally vacant i2 interstitial position (0.236, 0.014, 0.792) identified by SoftBV in the conduction mechanism (see Table S20) was simultaneously refined, then occupancies of 0.84(3) and 0.16(3) were obtained for the normal and interstitial Li sites in LiAlCl_4_ when the respective thermal parameters were fixed. By contrast, no evidence for antisite mixing (Li–Al disorder) was obtained for any of the halides. Although these results are not conclusive, they do tend to support observations from solid-state NMR spectroscopy of a partially occupied interstitial site in LiAlCl_4_^25^ and provide a rationale for the Li^+^ diffusion mechanism elucidated by BVSE and MD analyses for the haloaluminates (see below). It will be interesting to see whether local structural approaches, such as pair distribution function (PDF) analysis can provide further information regarding the links between defect structure and Li-ion motion.

The thermal stabilities of LiAlX_4_ were studied by simultaneous thermogravimetric-differential thermal analysis (TG-DTA). For all samples, the TG profiles are typical of thermal decomposition with volatile decomposition products (Figures S17–19). The decomposition is preceded by melting in each case (the melting points, as determined by the respective DTA peak onsets, are summarized in Table 1). The melting points of the tetrahaloaluminates increase from X = Cl through Br to I, with the new iodide, LiAlI_4_, melting at ∼509 K (and decomposing from ∼593 K). TG-DTA also confirmed the absence of unreacted AlX_3_ in the synthesized LiAlX_4_ materials with no AlX_*3*_ melting transitions visible in the DTA data (e.g., AlI_3_ melts at 461.43 K).^27^

**Table 1:** Thermal Properties and Transport Data of Mechanochemically Synthesized Lithium Tetrahaloaluminates

Material	Melting point (K)	Decomposition onset temperature (K)	σ_RT_ × 10^5^ (S cm^–1^)	σ_0_ × 10^–5^ (K S cm^–1^)	*E*_a_ (eV)
LiAlCl_4_	420.0 [419,^18^ 422^16^]	∼643	2.9(2) [2.1^25^]	8.6(7)	0.473(2) [0.47,^18^ 0.50(6)^25^]
LiAlBr_4_	464.9 [466^16^]	∼593	3.3(2)	2.5(4)	0.437(4)
LiAlI_4_	509.0	∼593	1.2(1)	0.61(7)	0.429(3)

The ionic transport properties of mechanochemically synthesized LiAlX_4_ were analyzed by variable temperature electrochemical impedance spectroscopy (EIS) with blocking Au electrodes. Figure 3a shows the room temperature Nyquist plots. For LiAlCl_4_, the spectrum was fitted with an equivalent circuit consisting of one parallel constant phase element (CPE)/resistor in series with a CPE representing the behavior of the electrolyte and blocking electrodes, respectively. The capacitance of the CPE/resistor is 2.5 × 10^–11^ F cm^–2^ with an α-value of 0.99, which together indicate a predominant bulk contribution to the impedance response.^28^ Conversely, the LiAlBr_4_ and LiAlI_4_ spectra were fitted with an equivalent circuit consisting of two parallel constant phase elements (CPE)/resistors in series with a further CPE. The capacitances of the high-frequency CPE/resistor elements are 2.4 × 10^–11^ (LiAlBr_4_) and 1.7 × 10^–11^ F cm^–2^ (LiAlI_4_) with α-values of 0.96 and 0.91, respectively, which represent the ideality of the CPE and confirm a bulk-process.

**Figure 3:**
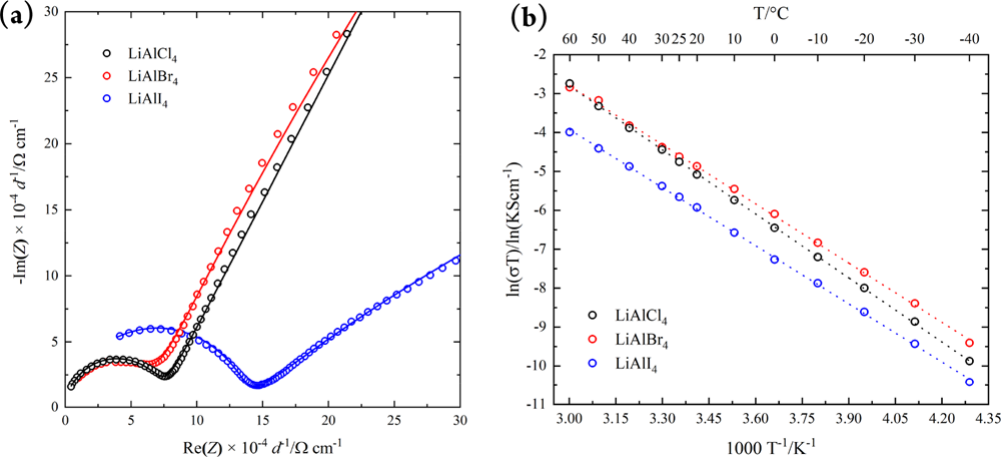
(a) Room-temperature Nyquist plots of LiAlX_4_ (X = Cl, Br, I) normalized to the thickness of the pellets, showing the impedance responses (open circles) and fits (solid lines). (b) Arrhenius plots of conductivity values obtained from temperature-dependent impedance spectroscopy.

At lower frequencies, impedance contributions are observed with capacitances of 2.5 × 10^–9^ (X = Br) and 8.1 × 10^–7^ F cm^–2^ (X = I). This can be attributed to a surface layer, which may be formed on thermal decomposition during gold coating, for example.^6,28^

Arrhenius behavior was noted for all samples in the LiAlX_4_ series across a temperature range of 233–333 K (Figure 3b). Room-temperature total ionic conductivities (σ_RT_) and the parameters extracted from linear fits of the Arrhenius plots (σ_0_, *E*_a_) are summarized in Table 1. We found that the room temperature Li^+^ conductivity first increases but then subsequently decreases when switching from X = Cl through Br to I. The results corroborate the premise that both the exponential prefactor, σ_0_, and the activation energy for Li^+^ diffusion decrease with increasing anion polarizability, although the differences in the *E*_a_ values for LiAlBr_4_ and LiAlI_4_ are not statistically significant.^29,30^ In this regard, it should be noted that a reduction in *E*_a_ does not always lead to improved ionic conductivity, since *E*_a_ and σ_0_ are correlated in line with the Meyer–Neldel rule.^31,32^ By considering both parameters in Table 1, it can be appreciated why LiAlI_4_ might have the lowest ionic conductivity within the haloaluminate series. It should be noted that the ionic conductivity for LiAlCl_4_ measured here is 1 order of magnitude higher than that reported by Weppner and Huggins.^18^ Although our mechanochemical syntheses are quicker and less energy-intensive to that recently reported for LiAlCl_4_ by Tanibata et al., the EIS data do further support the premise that ball milling positively influences the ionic conductivity in the lithium halides (likely facilitating defect formation compared to thermal synthesis methods, which are evidently not viable for X = I).^25^ Indeed, EIS measurements performed on a pellet of the LiAlCl_4_ sample that was subsequently annealed at 373 K for 14 h yielded a lower room temperature conductivity (of 1.2(2) × 10^–5^ S cm^–1^) than that of the cold-pressed mechanochemically synthesized chloride.

The comprehensive structural models obtained from neutron and synchrotron diffraction allowed the probable Li^+^ diffusion pathways in the haloaluminates to be established via bond-valence site energy (BVSE) analysis.^21^ In this class of materials, our analysis shows that the ionic conductivity is governed by the presence of intrinsic tetrahedral and octahedral interstitial sites. The BVSE map of LiAlI_4_ is presented as an example in Figure 4a, and shows that a bottleneck for 2D conduction involves Li^+^ hopping from its normal octahedral 4*e* lattice position to an adjacent tetrahedral 4*e* site (“i7” in the BVSE map notation in Figure 4b). Alternatively, 3D conduction requires hops from/to interstitial octahedral 4*e* lattice sites (“i3” → “i2”). Qualitatively, the BVSE models of the migration barriers in the LiAlX_4_ series depict very similar energy landscapes (while noting that the overall activation energies for LiAlBr_4_ and LiAlI_4_ are higher than the experimental values due to the level of accuracy of SoftBV, Figures 4b, S20, and S21). These energy profiles indicate that the conduction pathways do not change significantly with the halide and that the observed differences in ionic conductivity cannot be rationalized only via a static, crystal chemistry treatment.

**Figure 4:**
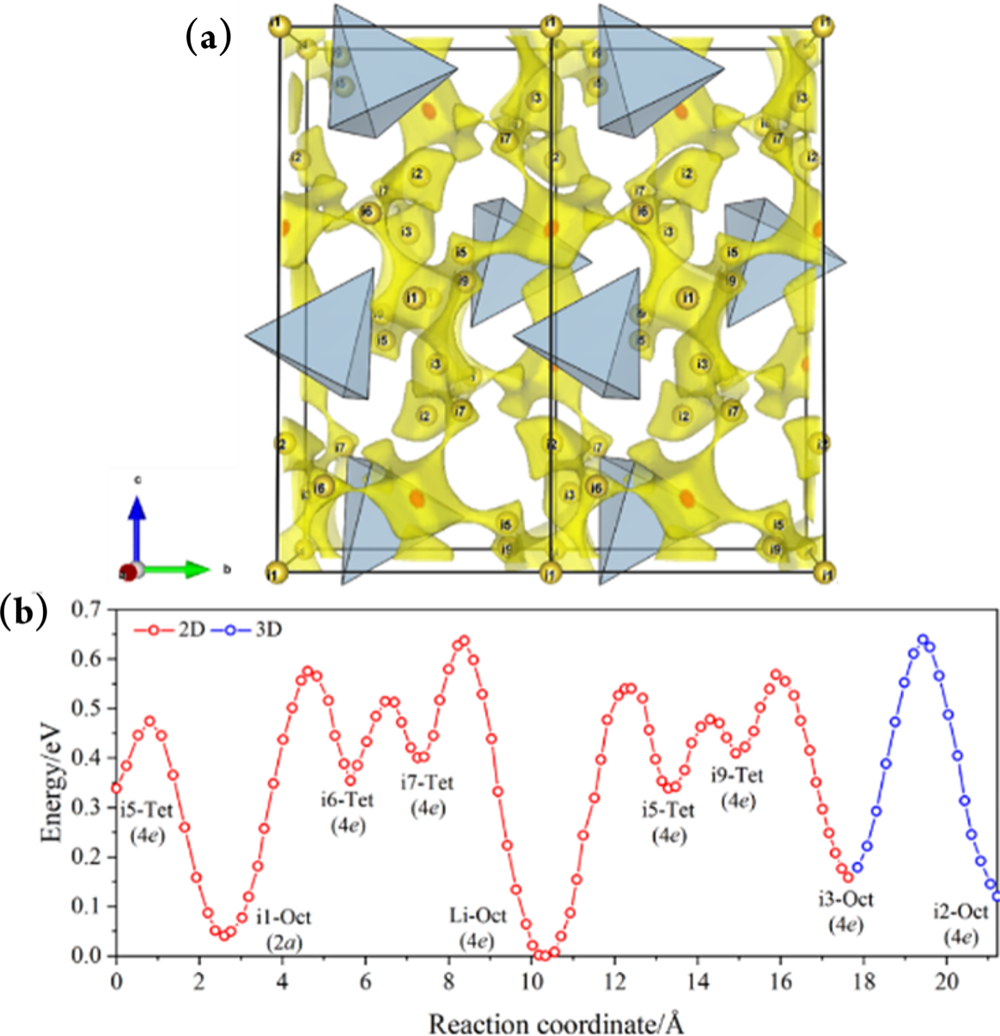
(a) BVSE map showing Li^+^ migration pathways in a (100) projection of the LiAlI_4_ structure, as visualized with VESTA.^26^ The highest isosurface level of 0.64 eV over the global minimum is shown in yellow. Red dots indicate octahedral Li^+^ lattice sites and yellow spheres indicate tetrahedral/octahedral interstitial sites. (b) BVSE model of migration barriers for LiAlI_4_ derived from Rietveld refinements against SPXD and PND data. The relative site energy is zero for Li^+^ lattice sites.

Empirical molecular dynamics simulations for a 768 atom 4 × 4 × 2 supercell of LiAlCl_4_ over the temperature range 250–400 K over 1500–18000 ps harmonize to the experimentally observed conductivity (Figure S22). A more detailed analysis shows that the 2D Li^+^ motion in the *y*–*z* plane is coupled to dynamic anion disorder (librations) that in the experimental study may be facilitated by the mechanochemical synthesis. Details are given in the Supporting Information (Figures S23 and S24). Given the role of polyanion motions in other cation conductors, the combination of experimental and computational data encourages further investigations of the role of defects and dynamic anion effects in LiAlX_4_ materials and how such effects might be tuned.

In view of the promising ionic transport behavior, preliminary linear sweep voltammetry (LSV) experiments were conducted to determine the oxidative stability of LiAlX_4_. InLi and a carbon + SSE composite were employed as the counter and working electrodes, respectively. The oxidative stability limits were defined by the onset potentials (*E*_onset_) and calculated via linear fitting of the nonfaradaic and faradaic region.^33^ The room temperature voltammograms are shown in Figure 5.

**Figure 5:**
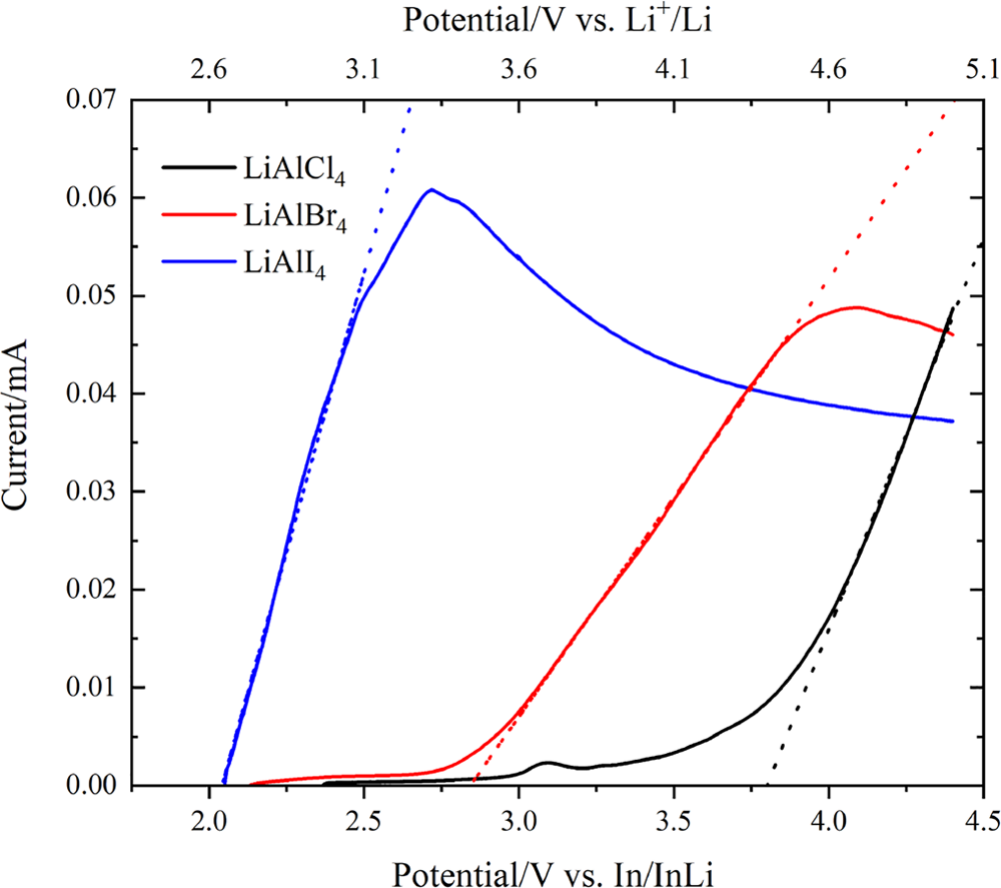
Room temperature linear sweep voltammogram (0.1 mV s^–1^) of InLi|LiAlX_4_|LiAlX_4_ + C cells. Dashed lines indicate linear fits of the faradaic region. The bottom *x*-axis shows the values of the voltage versus In/InLi, the top *x*-axis shows the corresponding values of voltage versus Li^+^/Li.^34^

We determined that oxidation of LiAlX_4_ materials starts at 3.8 (4.4), 2.8 (3.4), and 2.0 (2.6) V versus In/InLi (vs Li^+^/Li) for X = Cl, Br, and I, respectively. Density functional theory (DFT) calculations predicted the electrochemical windows of LiAlX_4_ to be either 1.7–4.5 V^35^ or 1.54–4.45 V^7^ for X = Cl and 1.8–3.9 V^35^ (vs Li^+^/Li), for X = Br. Above the oxidation limits, LiAlX_4_ are predicted to produce AlX_3_ and X_2_, while below the reduction limits, LiAlX_4_ are predicted to form Al and LiX, indicating that lithium tetrachloro- and tetrabromoaluminates are not stable against Li metal, as is true for the liquid electrolyte, LiAlCl_4_·3SO_2_.^36^ These results indicate the feasibility of combining LiAlCl_4_ with high voltage cathode materials, while the bromide and iodide analogues would be better suited to use with lower potential electrodes, such as sulfide-based cathodes, for potential cell applications. Equally, use of LiAlBr_4_ and LiAlI_4_ with high voltage cathodes might be enabled with an appropriate coating of the positive electrode to prevent SSE decomposition.^37,38^ Further investigations into various half- and full-cell architectures and their performance are currently underway and will be reported elsewhere.

In summary, we have shown that high purity lithium tetrahaloaluminate powders can be easily synthesized by mechanochemical methods. This approach is extremely effective in preparing bulk quantities of LiAlX_4_ including the new iodide, LiAlI_4_, which could not be synthesized by thermal methods. Synchrotron and neutron diffraction have shown not only that the bromide and iodide are isostructural to the chloride analogue but that Li vacancies and interstitials are likely prevailing features of mechanically synthesized tetrahaloaluminates. Each material exhibits appreciable Li-ionic conductivity, good thermal stability and reasonable stability to oxidation, such that the chloride, especially, might be employed with high voltage cathodes. Moreover, Li^+^ conductivity and stability can likely be improved still further by tuning the microstructure, composition and defect chemistry of the haloaluminates through doping, substitution and compositing. The LiAlX_4_ family suggests these and other polyanion halides offer considerable promise as new classes of cation conductor and as candidates for testing systematically as SSEs.

## Supporting Information Available

Experimental procedures, characterization techniques, crystallographic information, TG-DTA profiles, BVSE analysis, and details of MD simulations (PDF)
