# Supplementary material for: Mechanochemical Synthesis and Structure of Lithium Tetrahaloaluminates, LiAlX4 (X = Cl, Br, I): A Family of Li-Ion Conducting Ternary Halides
Source: ACS Mater Lett. 2021 Apr 20;3(5):652–7. doi: 10.1021/acsmaterialslett.1c00055 (PMC8397468; doi:10.1021/acsmaterialslett.1c00055)
Supplement: Supplementary file 1 — tz1c00055_si_001.pdf [file tz1c00055_si_001.pdf]

Mechanochemical synthesis and structure of lithium tetrahaloaluminates, $LiAlX_4$ (X = Cl, Br, I); a family of Li-ion conducting ternary halides.

*Nicolás Flores-González, Nicolò Minafra, Georg Dewald, Hazel Reardon, Ronald I. Smith, Stefan Adams, Wolfgang G. Zeier and Duncan H. Gregory**

Nicolás Flores-González, Dr. Hazel Reardon[1], Prof. Duncan H. Gregory
School of Chemistry, Joseph Black Building
University of Glasgow
Glasgow, G12 8QQ, United Kingdom
E-mail: duncan.gregory@glasgow.ac.uk

Georg Dewald
Institute of Physical Chemistry
Justus-Liebig-University Giessen
Heinrich-Buff-Ring 17, D-35392 Giessen, Germany

Dr. Ronald I. Smith
ISIS Pulsed Neutron and Muon Source
STFC Rutherford Appleton Laboratory
Didcot, Oxfordshire, OX11 0QX, United Kingdom

Prof. Stefan Adams
Department of Materials Science and Engineering
National University of Singapore
9 Engineering Drive 1, 117575, Singapore

Nicolò Minafra, Prof. Wolfgang G. Zeier
Institute for Inorganic and Analytical Chemistry
University of Munster
Correnstr 30, 48149, Germany

Experimental procedures

Synthesis of $LiAlX_4$ (X = Cl, Br, I): LiCl (Sigma Aldrich, ≥ 99.99%), $AlCl_3$ (Sigma Aldrich, anhydrous, 99.999%), LiBr (Alfa Aesar, anhydrous, 99.995%), $AlBr_3$ (Sigma Aldrich, anhydrous, 99.999%), LiI (Sigma Aldrich, anhydrous, 99.99%) and AlI_3 (Sigma Aldrich, anhydrous, 99.999%) were used as starting materials without further purification. Because of the air and/or moisture sensitivity of the starting and the final product, all manipulations were carried out in a recirculating Ar-filled (BOC, 99.998%) glove box (MBraun LABstar, O_2 < 0.5 ppm, H_2O < 0.5 ppm). *Ca.* 0.5 g in total of the starting materials were weighed out accurately in the desired molar ratio (1:1) and then transferred

[1] Present address is at the Center for Materials Crystallography
Department of Chemistry and Interdisciplinary Nanoscience Center (iNANO)
Aarhus University
8000-DK Aarhus (Denmark)

to a stainless-steel grinding jar that was filled with either ten or six stainless steel balls (10 mm diameter), each of which weighed *ca.* 4 g. The grinding jar was sealed under Ar before removal from the glove box. Ball milling was conducted with a ball-to-powder ratio (BPR) of 80:1 in a planetary ball mill (Retsch PM100) in 5 minute milling periods (reverse rotation), which were followed by 5 minute rest periods. Both speed and milling time were optimized in order to obtain single phases (Table S1). The optimised samples used for subsequent characterisation and measurements are listed in Table S2.

$LiAlCl_4$ – Synthesis usually involves heating of the corresponding anhydrous salts, LiCl and $AlCl_3$ (*e.g.* at 150 °C for *ca.* 1 h) but other authors have used more laborious methods such as reacting LiCl and $AlCl_3$ in liquid SO_2 at -10 °C, which requires subsequent elimination of the solvent.[1,2] In our mechanochemical approach, we fixed the speed to 450 rpm and prepared samples with gradually increased milling times from 1 to 5 hours. After as little as 1 h, peaks assigned to $LiAlCl_4$ were observed (Figure S1a), however, reflections corresponding to LiCl were also evident, suggesting that the reaction was incomplete. On increasing the milling time from 1 to 3 h, reflections assigned to LiCl were negligible, indicating reaction completion (Figure S1b). Conversely, after milling for 5 h, the peaks corresponding to $LiAlCl_4$ broadened significantly, suggesting the onset of amorphization in the final product (Figure S1c). Thus, the optimum milling parameters to achieve a crystalline single-phase product, which could be reliably characterized, were set to 450 rpm/3 h (sample **2**).

$LiAlBr_4$ – In 2015, Scholz *et al.*[2] demonstrated that crystals of $LiAlBr_4$ could be grown from the melt by heating the corresponding anhydrous salts, LiBr and $AlBr_3$ at 200 °C for *ca.* 1 h. We could not easily employ this method to make bulk quantities of phase-pure powders however, so our first attempts to make $LiAlBr_4$ *via* ball milling were performed using the same parameters employed for $LiAlCl_4$ (sample **2,** Table S1), with gradually increased milling times from 3 to 9 h. The reaction progress was analysed by following the peak intensity of LiBr at $2\theta = 32.53°$ (Figure S2). After milling for 3 h only, peaks assigned to $LiAlBr_4$ were observed. However, LiBr was detected in all patterns despite the increase in the milling time. Moreover, when increasing the milling time, an unidentified peak at $2\theta = 28.36°$ became more intense, which indicates an alternative reaction pathway and suggests that a lower energy input was needed. In a second attempt, the rotation speed was decreased and kept constant at 300 rpm, while the milling time was gradually increased from 4 to 8 h (Figure S3) Likewise, after milling for 4 h only, peaks assigned to $LiAlBr_4$ were observed. However, using these parameters, the peak intensity of LiBr at $2\theta = 32.53°$ decreased significantly. After milling for 6 h, the LiBr peak intensity is negligible, while on milling for 8 h, the LiBr peak intensity once more increases. These findings suggest that at 4 h the reaction was incomplete, but at 8 h the desired product had begun to decompose. Two reaction pathways have been proposed for the thermal decomposition of tetrahaloaluminates,[2, 3]

$$LiAlX_4 \rightarrow LiX + AlX_3 \quad (1)$$

$$2LiAlX_4 \rightarrow LiX + LiAl_2X_7 \quad (2)$$

Scholz *et al.*[2] demonstrated that the latter (Equation 2) is not preferred under thermodynamic equilibrium. However, those results may not be applicable to mechanochemical processes as they typically occur under non-equilibrium conditions and, therefore, metastable compounds can be formed.[4]

$LiAlI_4$ – We performed thermogravimetric-differential thermal analysis (TG-DTA) experiments (see procedure below) in order to study the feasibility of making $LiAlI_4$ *via* traditional solid-state synthesis. The available phase diagram of the binary system AlI_3-LiI shows the formation of a stoichiometric compound, $LiAlI_4$, which melts congruently *ca.* 240 °C.[5] Our thermal analysis showed that under the conditions studied, AlI_3 melts at 188.0 °C followed by decomposition at *ca.* 210 °C (Figure S4). Thus, the reaction between AlI_3 and LiI should be performed below the latter temperature in order to avoid thermal decomposition of the starting materials.

A 1:1 mixture of LiI:AlI_3 was preheated at 100 °C for 1 h and subsequently cooled to 50 °C in order to eliminate possible water traces present in the system. The dry mixture was then heated to 160 °C for 1 h before cooling to room temperature (all heating/cooling rates were set at 5 °C min^{-1}). An endothermic peak was observed at 159.9 °C attributed to the reaction between AlI_3 and LiI, followed by an almost immediate mass loss of 2.2%. During cooling, a second compound seemed to consolidate at 147.7 °C indicating an incomplete reaction in agreement with PXD data (Figures S5,6). These observations indicate that phase-pure $LiAlI_4$ cannot be easily isolated thermally and, therefore, a softer synthetic method was considered.

First attempts to make $LiAlI_4$ *via* ball milling were performed using identical experimental parameters to those employed in the preparation of $LiAlBr_4$ (sample **8**, Table S1) and by increasing the milling time sequentially from 1.5 h to 6 h. After milling for 1.5 h, a new poorly crystalline phase was observed. Neither of the starting materials LiI and AlI_3 were detected as crystalline phases. Increasing the milling time resulted in a further decrease of crystallinity (Figure S7). These findings suggested that either the new phase was inherently disordered/glassy or that the ball milling parameters were too harsh, prompting amorphisation. To asses these hypotheses, the rotation speed was decreased from 300 to 200 rpm. Results are shown in Figure S8. After milling for 1.5 h at 200 rpm, the powder pattern of the product closely resembled that from milling at 300 rpm for 1.5 h. However, the crystallinity of the product improved considerably with prolonged milling time (200 rpm for 3 h or 6 h). A similar effect was also observed by Katsenis *et al.*[6] when they monitored the mechanochemical synthesis of a metal organic framework *in situ* using X-ray diffraction.

Characterisation Techniques

Powder X-Ray diffraction (PXD): Powder X–ray diffraction experiments were conducted at room temperature with a Bruker D8 Advance diffractometer in transmission geometry using Cu Kα radiation (1.54184 Å) with a slit size of 2.0 mm. The moisture/air-sensitive samples were loaded inside glass capillaries (0.5 or 0.7 mm internal diameter) and sealed with wax in an Ar-filled recirculating glovebox. The sample capillaries were flame-sealed outside the glove box. The aligned capillaries were continuously rotated throughout the analysis and scanned over $5 \leq 2\theta / ° \leq 110$ ranges (0.0167 step size, 14-15 h).

Powder Synchrotron X-Ray diffraction (SPXD): In order to avoid absorption, powdered samples were loaded inside 0.3 mm glass capillaries. SPXD patterns were collected using the Material Science Beamline X04SA at the Paul Scherrer Institute (PSI) in Switzerland.[7] A Mythen II detector was used to collect data in the range of $1 \leq 2\theta / ° \leq 90$ (0.0036° step size, 1 h) in transmission mode using a 22 keV X-ray beam at room temperature. The exact wavelength was refined from the lattice parameters of a Si 640d NIST standard, while the instrument parameters were obtained from the refinement of a $Na_2Ca_3Al_2F_{14}$ (NAC) standard.

Time-of-flight powder neutron diffraction (PND): Data were collected on the Polaris powder diffractometer at the ISIS pulsed spallation neutron source, Rutherford Appleton Laboratory, UK.[8] ~1 g of powder was loaded into a 6 mm or 8 mm diameter thin-walled vanadium sample can, which was sealed using indium wire, inside a glove box. The sample cans were mounted in the diffractometer and data collected at room temperature for a duration of 170 μA h integrated proton beam current to the ISIS neutron target (corresponding to ~1 h total exposure). Data reduction, which included an empirical absorption correction, and generation of files suitable for profile refinement, used the Mantid open source software.[9]

Rietveld analysis: Structure refinement was carried out by the Rietveld method[10], employing GSAS-EXPGUI[11, 12] and GSAS-II[13] software for PND and SPXD data, respectively. R_{wp} and χ^2 fit indicators were used to assess the quality of the refined structural model.[14] The refinements were conducted in consecutive steps as follows: background (using the reciprocal interpolation and cosine functions for PND and SPXD data, respectively), scale factors, unit cell parameters, peak width parameters, fractional atomic coordinates (Li fractional atomic coordinates obtained from the refinements against PND data were used and fixed in the refinements against SPXD data), (an)isotropic atomic displacement parameters and atomic site occupancies. In consideration of the NMR data published recently for mechanochemically-synthesised $LiAlCl_4$[15], we assessed models in which the

interstitial tetrahedral sites revealed by softBV (see Table S20) were partially occupied. Among them, when the initial i2 interstitial position at (0.236, 0.014, 0.792) was simultaneously refined, then occupancies of 0.84(3) and 0.16(3) were obtained for the normal and interstitial Li sites, respectively. The refined coordinates for the i2 site are (0.21(2), 0.89(2), 0.806(8)). However, attempted simultaneous refinement of its atomic displacement parameter (set to an initial value of 0.05 Å^2) led to negative values. Conversely, for the bromide and iodide analogues, attempted refinement of interstitial tetrahedral sites revealed by softBV (Tables S21,2) resulted into either unstable refinements or unphysical values (*e.g.* negative U_{iso} values). Based on the above results, in our final models, all atomic site occupancies were fixed to 1. Specifically, for the PND data, refinements were performed using multiple banks: $LiAlCl_4$ (banks 5 and 4), $LiAlBr_4$ (banks 5-3) and $LiAlI_4$ (banks 5-2). In all PND refinements, vanadium peaks (originating from the sample cans) were fitted as a secondary phase (not shown in the profile fits).

Thermogravimetric-Differential Thermal Analysis (TG-DTA): The thermal stability of all samples was measured by simultaneous thermogravimetric-differential thermal analysis using a Netzsch STA 409 instrument contained within an Ar-filled MBraun UniLab recirculating glovebox (O_2 and H_2O <0.1 ppm). Accurately weighed samples of 15-30 mg were heated in alumina crucibles under a constant flow of Ar (BOC, ≥ 99.999%, 60 mL min^{-1}) from 30 °C to the desired target temperature at a 5 °C min^{-1} heating rate.

Electrochemical Impedance Spectroscopy (EIS): Electrical conductivities were measured by AC EIS using isostatically pressed pellets (325 MPa, measured densities were 88, 84 and 80 % of the crystallographically determined values for *X* = Cl, Br, I, respectively), which were subsequently coated *via* thermal evaporation with thin gold (200 nm) electrodes. Electrochemical impedance analysis was conducted in the temperature range of -40 to 60 °C using a SP300 impedance analyser (Biologic) at frequencies from 7 MHz to 50 mHz with an amplitude of 10 mV.

Linear Sweep Voltammetry (LSV): Press cells were employed for LSV experiments. First, 100 mg of the SE material ($LiAlX_4$) was filled into the PEEK housing and manually compressed with stainless steel stamps. For the SSE+C electrode composite, the respective SSE and carbon (Super C65, TIMCAL) were mixed in a weight ratio of 9:1 and manually ground in an agate mortar for 15 min. A mass of 15 mg of SSE+C composite was put onto the SSE pellet and was uniaxially pressed for 3 min at 35 kN. The In-Li electrode was obtained by pressing together a piece of In (9 mm diameter, 100 μm thick, ChemPur, 99.999%) and Li foil (6 mm diameter, 120 μm thick, Sigma Aldrich 99.9%), which was subsequently added to the opposite face of the pellet. Finally, the cell was fixed in an aluminium frame with a torque of 10 Nm, resulting in a pressure of approximately 60 MPa. After assembly, each cell was allowed to equilibrate for 1 h to ensure good alloying at the counter/reference electrode and to establish

a stable open-circuit voltage (OCV) prior to any measurements. Therefore, all voltammetry data are reported vs. In/InLi. The LSV experiments were performed using a VMP300 potentiostat (Biologic) at a constant temperature of 25 °C and a scan rate of 0.1 mV s^{-1}. Each experiment started at the OCV and the potential was swept to 4.4 V vs. In/InLi.

Molecular Dynamics (MD) Simulations: Starting from the experimental crystal structure of $LiAlCl_4$ refined in this work, we created a 4×4×2 supercell containing 768 atoms and adapted our bond-valence-site energy forcefield to reproduce the crystal structure with a pressure < 0.3 GPa in NVT simulations maintaining the experimental lattice parameters. All MD simulations were conducted with the GULP simulation software.[16] Compared to the standard two-body softBV forcefield, here we added EAM-inspired multibody parameters to ensure that the bond valence sum deviation remains small throughout the simulation and a three-body term to enhance the rigidity of the $[AlCl_4]^-$ tetrahedra. The resulting forcefield (see Table S23) was employed to conduct MD simulations at temperatures over the range 250 K to 400 K over 1500 to 18 000 ps in time steps of 1-1.5 fs. The limiting slopes of the mean squares displacement have then be converted to ionic conductivities assuming validity of the Nernst-Einstein relationship. For the temperature range 250-375 K an effective activation energy of 0.47(2) eV with a $\sigma(300\text{ K}) \approx 9\times10^{-5}$ S cm^{-1} is found in close harmony to the experimentally observed MD simulation (Figure S22). As already predicted by the bond valence site energy calculations, the Li^+ ions migrate predominantly in the *y*-*z* plane with only marginal local mobility in the *x* direction up to 350 K (Figure S23). Visual inspection of the trajectory files reveals (especially for the lower temperatures, where the total number of Li^+ hops is limited) that Li^+ hops predominantly occur directly after reorientations of the $[AlCl_4]^-$ tetrahedra, as exemplified in the snapshot details in Figure S24.

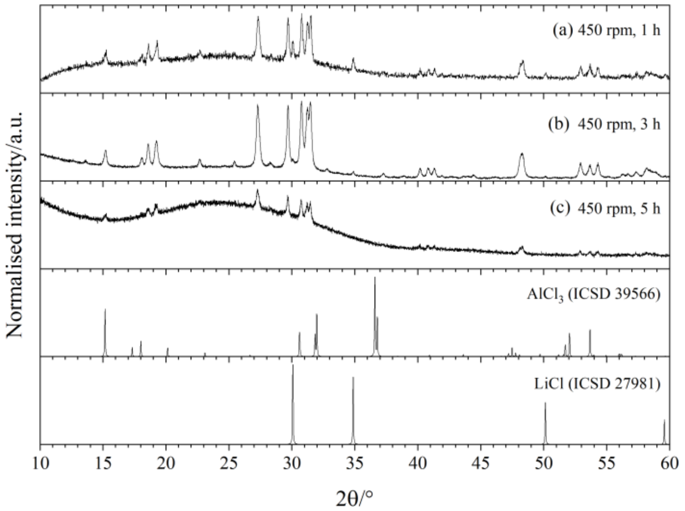

Figure S1. Room temperature PXD patterns of 1:1 LiCl:$AlCl_3$ after milling at: (a) 450 rpm, 1 h; (b) 450 rpm, 3 h; (c) 450 rpm, 5 h as compared to the diffractions patterns of the respective starting materials generated from the ICSD database. The optimum milling parameters to prepare crystalline single-phase $LiAlCl_4$ are 450 rpm/3 h.

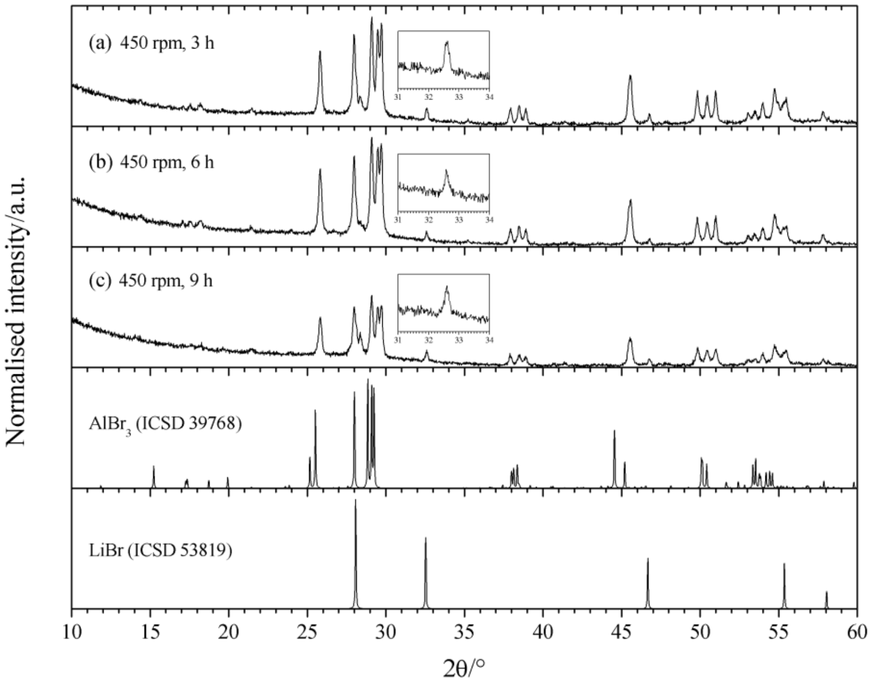

Figure S2. Room temperature PXD patterns of 1:1 LiBr:$AlBr_3$ after milling at: (a) 450 rpm, 3 h; (b) 450 rpm, 6 h; (c) 450 rpm, 9 h as compared to the diffractions patterns of the respective starting materials generated from the ICSD database. Under these milling parameters, a peak at 2θ = 32.53°, corresponding to LiBr (inset), was detected in all patterns despite the increase in the milling time.

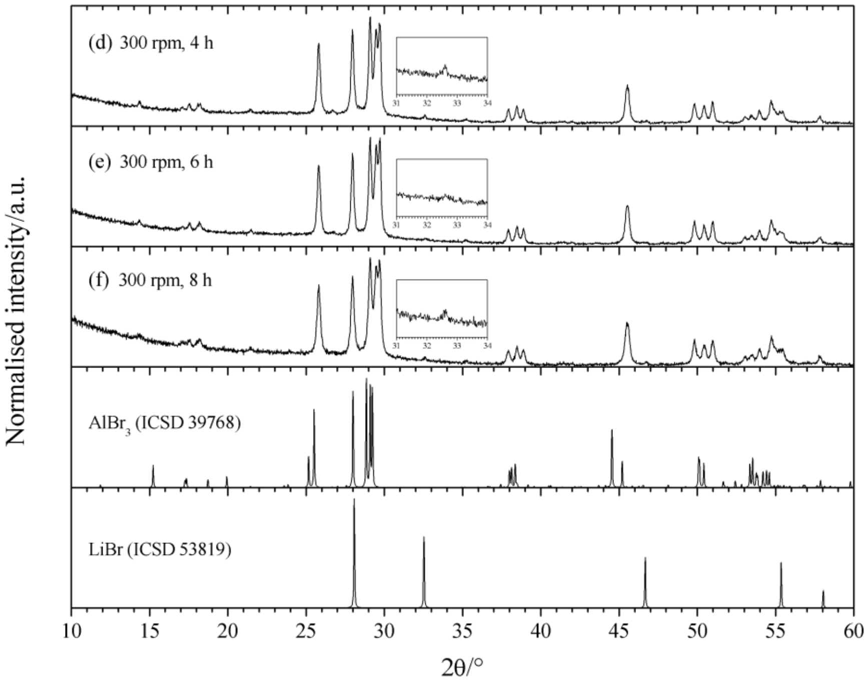

Figure S3. Room temperature PXD patterns of 1:1 LiBr:AlBr$_3$ after milling at: (d) 300 rpm, 3 h; (e) 300 rpm, 6 h; (f) 300 rpm, 9 h as compared to the diffractions patterns of the respective starting materials generated from the ICSD database. By decreasing the rotation speed from 450 to 300 rpm, the peak intensity at 2θ = 32.53°, corresponding to LiBr (inset), decreased significantly and it was found to be negligible after milling for 6 h.

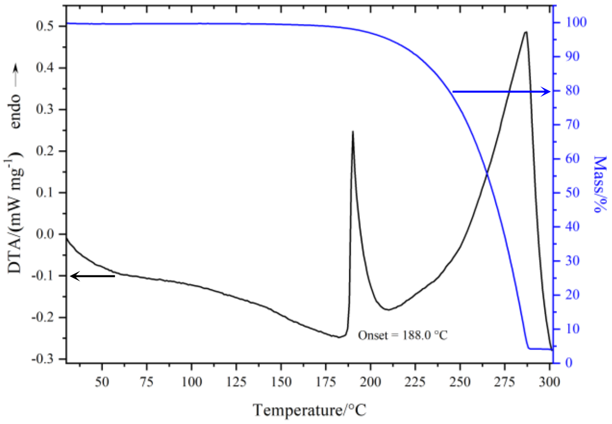

Figure S4. TG-DTA profiles of AlI$_3$ heated to 300 °C at 5 °C min^{-1} under flowing Ar (60 mL min^{-1}). The endotherm at 188.0 °C is attributed to the melting of AlI$_3$. Decomposition occurs from *ca.* 210 °C.

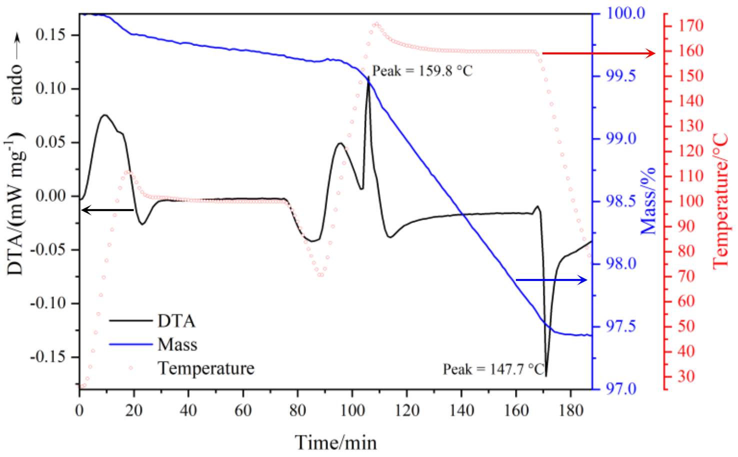

Figure S5. TG-DTA profiles of a 1:1 LiI:AlI_3 mixture preheated at 100 °C for 1 h and subsequently cooled to 50 °C. The dry mixture was then heated to 160 °C for 1 h before cooling to room temperature (all heating/cooling rates were set at 5 °C min^{-1} under flowing Ar at 60 mL min^{-1}). An endothermic peak was observed at 159.9 °C attributed to the reaction between AlI_3 and LiI, followed by an almost immediate mass loss of 2.2%. During cooling, a compound appeared to consolidate at 147.7 °C.

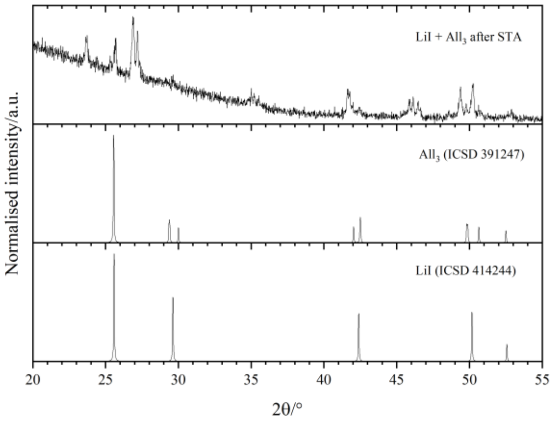

Figure S6. Room temperature PXD patterns of 1:1 LiI:AlI_3 after the TG-DTA experiment (shown in Figure S5) as compared to the diffractions patterns of the respective starting materials generated from the ICSD database.

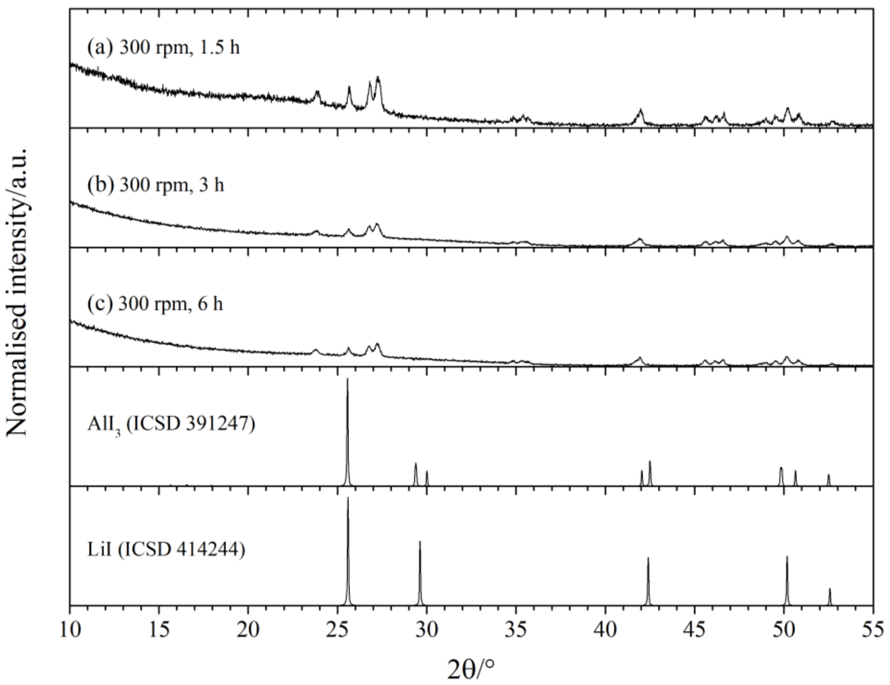

Figure S7. Room temperature PXD patterns of 1:1 LiI:AlI_3 after milling at: (a) 300 rpm, 1.5 h; (b) 300 rpm, 3 h; (c) 300 rpm, 6 h as compared to the diffractions patterns of the starting materials generated from the ICSD database. Under these conditions, the formation of a new, poorly crystalline phase was observed. Neither of the starting materials LiI and AlI_3 were detected as crystalline phases. Increasing the milling time resulted in a further decrease of crystallinity.

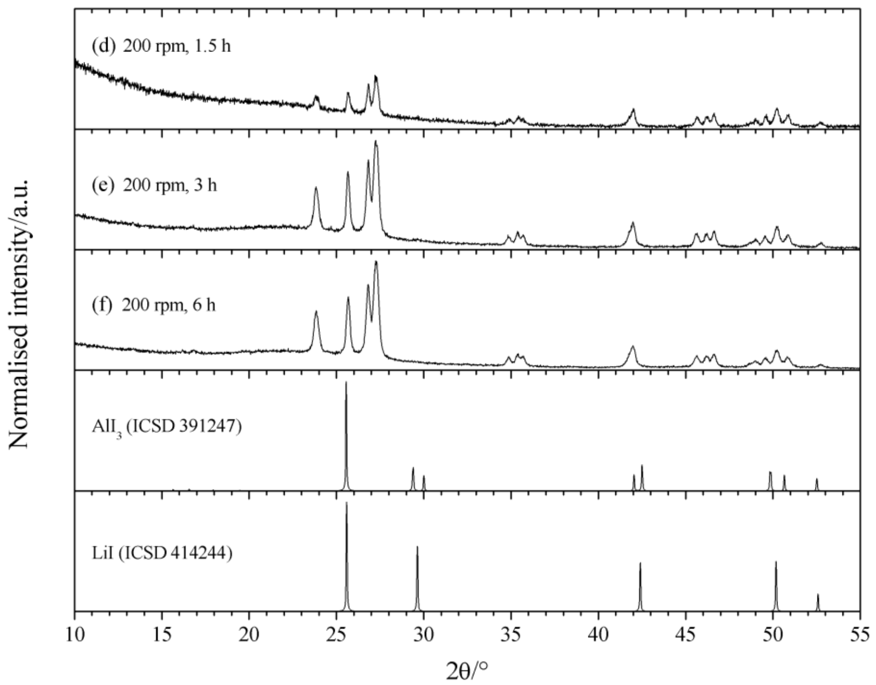

Figure S8. Room temperature PXD patterns of 1:1 LiI:AlI_3 after milling at: (d) 200 rpm, 1.5 h; (e) 200 rpm, 3 h; (f) 200 rpm, 6 h as compared to the diffractions patterns of the starting materials generated from the ICSD database. By decreasing the rotation speed from 300 to 200 rpm, the crystallinity of the product improved considerably as milling time was extended (to 3 or 6 h).

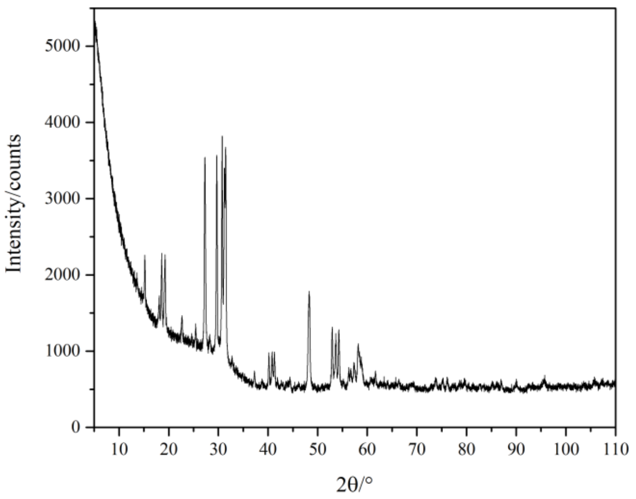

Figure S9. Room temperature PXD pattern of $LiAlCl_4$ (sample **2**).

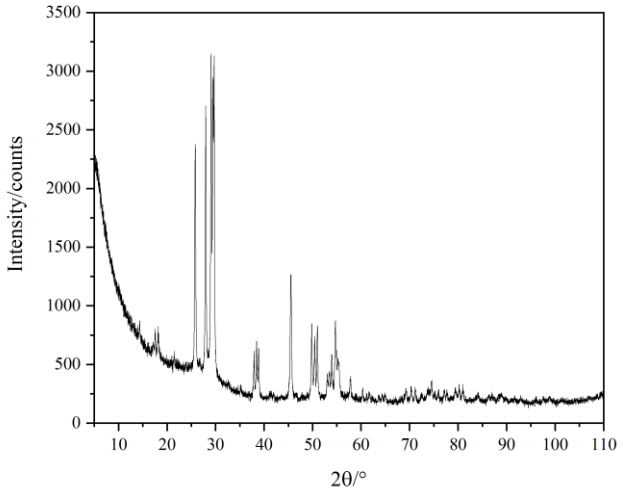

Figure S10. Room temperature PXD pattern of $LiAlBr_4$ (sample **8**).

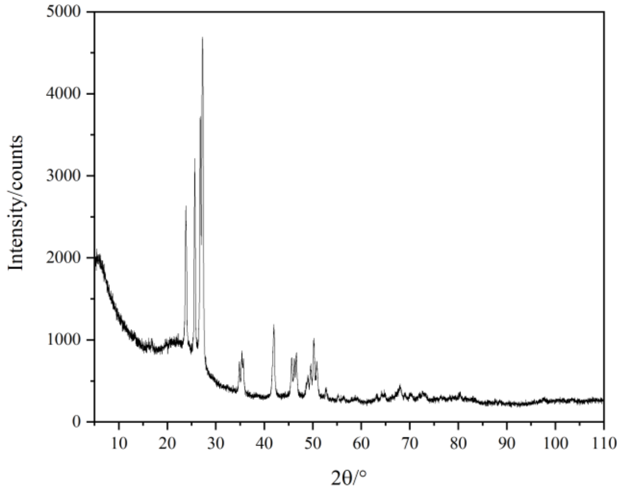

Figure S11. Room temperature PXD pattern of $LiAlI_4$ (sample **15**).

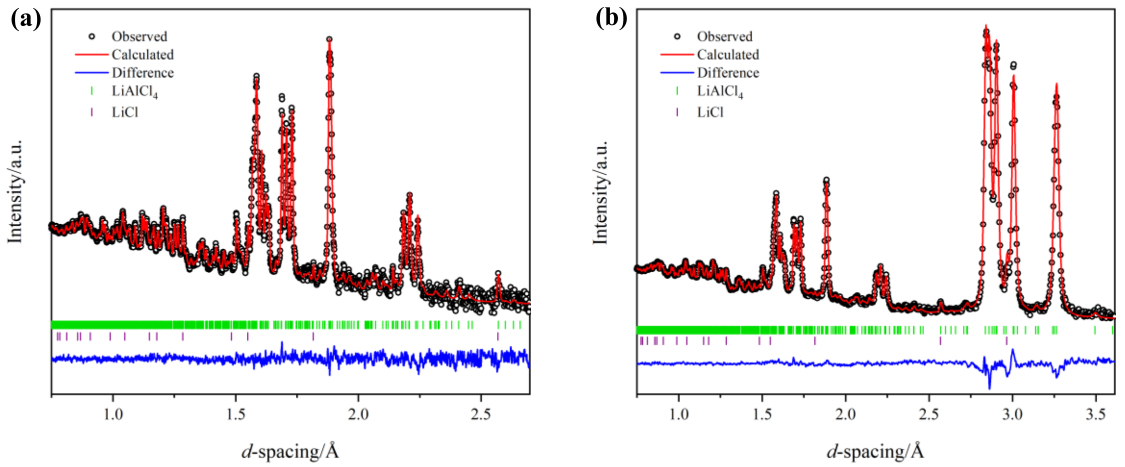

Figure S12. Room temperature profile fits from Rietveld refinement against time-of-flight powder neutron diffraction data for $LiAlCl_4$ (sample **2**), collected at Polaris, ISIS[8, 17]. **(a)** detector bank 5, (<2θ = 146.72°>), R_{wp} = 1.66 %, R_p = 2.64 %; **(b)** detector bank 4, (<2θ = 92.59°>), R_{wp} = 1.45 %, R_p =2.60 %. χ^2 = 1.09.

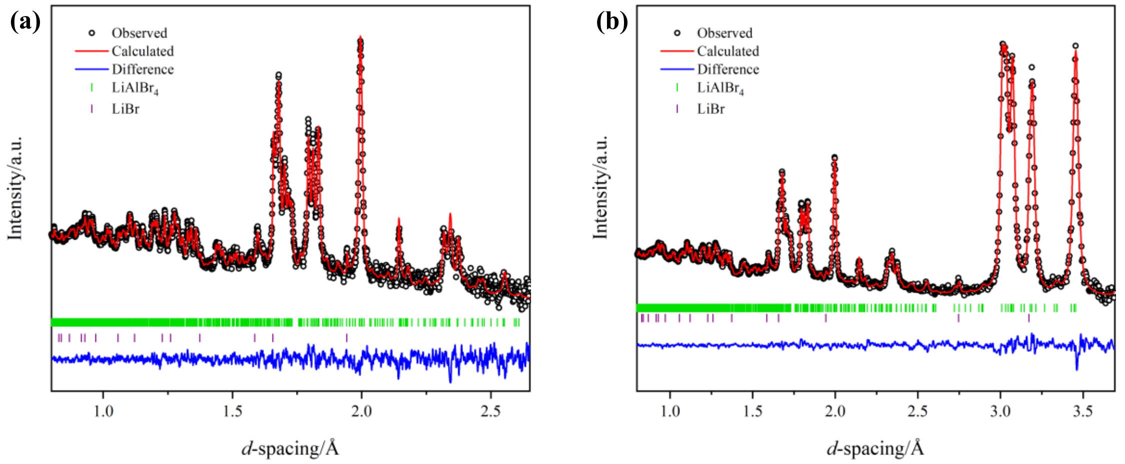

Figure S13. Room temperature profile fits from Rietveld refinement against time-of-flight powder neutron diffraction data for $LiAlBr_4$ (sample **8**) collected at Polaris, ISIS[8, 17]. **(a)** detector bank 5, (<2θ = 146.72°>), R_{wp} = 1.97 %, R_p = 2.87 %; **(b)** detector bank 4, (<2θ = 92.59°>), R_{wp} = 1.42 %, R_p = 2.25 %. χ^2 = 1.35.

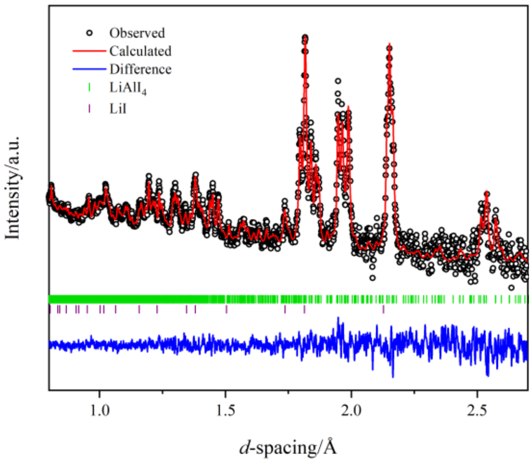

Figure S14. Room temperature profile fits from Rietveld refinement against time-of-flight powder neutron diffraction data for $LiAlI_4$ (sample **15**) collected at Polaris, ISIS[8, 17]. Detector bank 5, (<2θ = 146.72°>), R_{wp} = 1.62 %, R_p = 2.71 %.

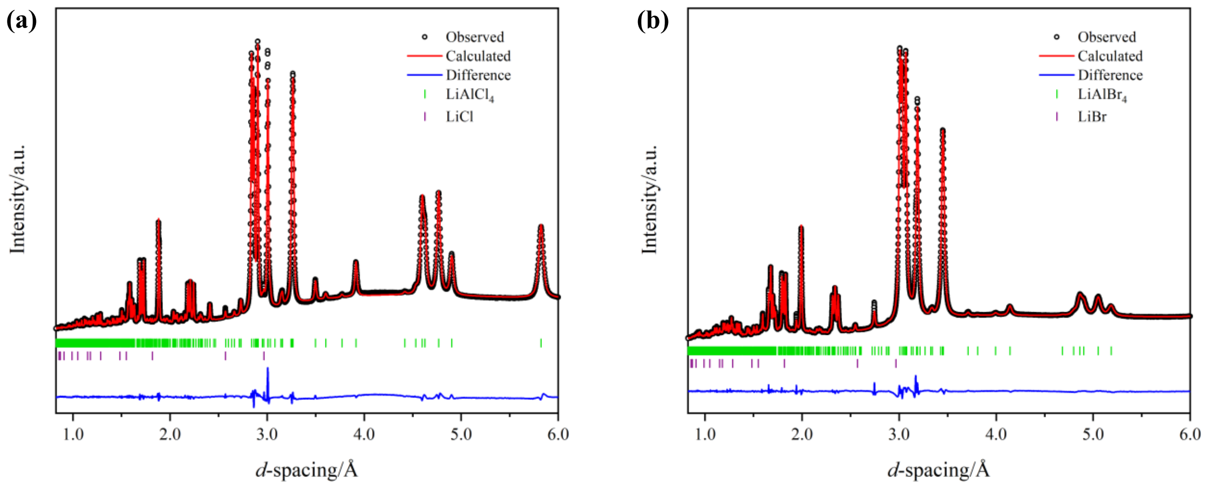

Figure S15. Room temperature profile fits from Rietveld refinement against Synchrotron powder X-ray diffraction data (λ = 0.56466 Å; X04SA, PSI) for **(a)** $LiAlCl_4$ (sample **2**), R_{wp} = 2.28 % χ^2 = 5.01; **(b)** $LiAlBr_4$ (sample **8**), R_{wp} = 2.07 % χ^2 = 4.42.

(a)

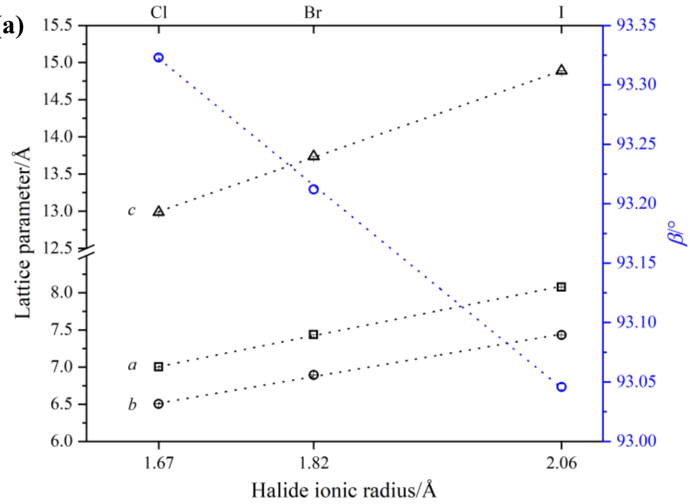

(b)

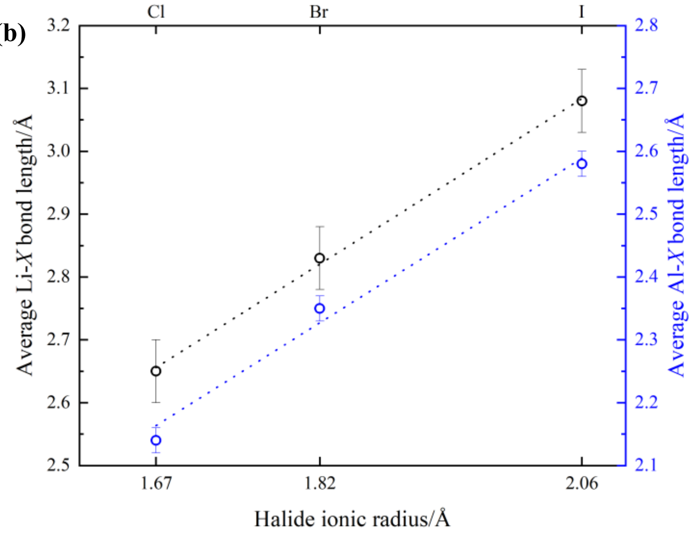

Figure S16. Plots of **(a)** lattice parameters (*a*, *b*, *c* and *β*) and **(b)** mean Li-*X* and Al-*X* bond lengths vs. halide ionic radius (with distances obtained from PND refinement data).

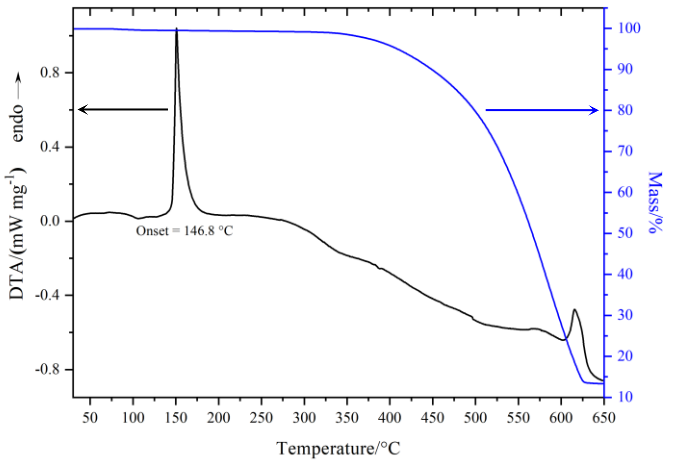

Figure S17. TG-DTA profiles of $LiAlCl_4$ (sample **2**) heated to 650 °C at 5 °C min^{-1} under flowing Ar (60 mL min^{-1}). The endotherm at 146.8 °C is attributed to the melting of $LiAlCl_4$. Decomposition begins at *ca.* 370 °C.

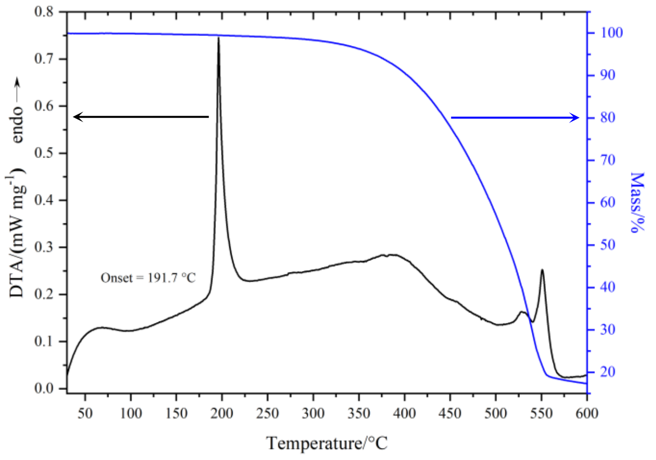

Figure S18. TG-DTA profiles of $LiAlBr_4$ (sample **8**) heated to 600 °C at 5 °C min^{-1} under flowing Ar (60 mL min^{-1}). The endotherm at 191.7 °C is attributed to the melting of $LiAlBr_4$. The decomposition onset is at *ca.* 320 °C.

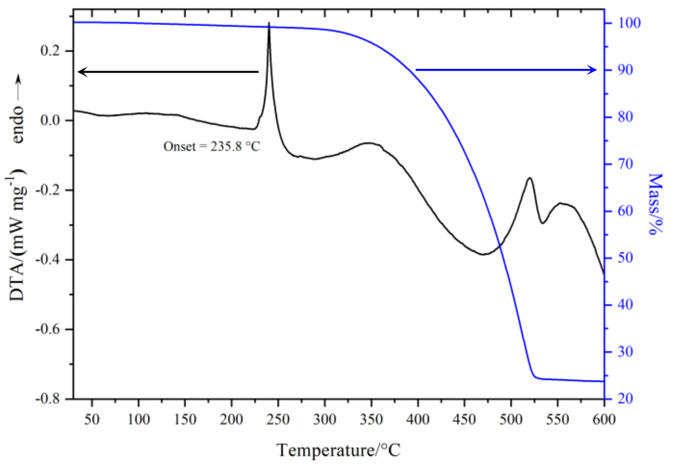

Figure S19. TG-DTA profiles of $LiAlI_4$ (sample **15**) heated to 600 °C at 5 °C min^{-1} under flowing Ar (60 mL min^{-1}). The endotherm at 235.8 °C is attributed to the melting of $LiAlI_4$. Decomposition occurs from *ca.* 320 °C.

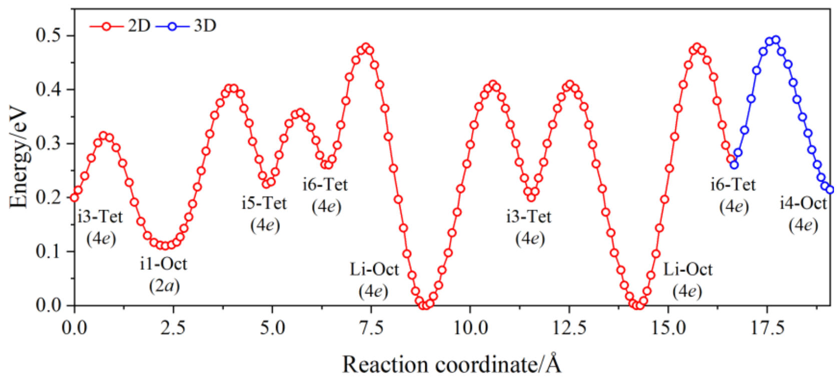

Figure S20. BVSE model of migration barriers for $LiAlCl_4$ using the models taken from the SPXD and PND data. The relative site energy is referred to zero for Li^+ lattice sites.

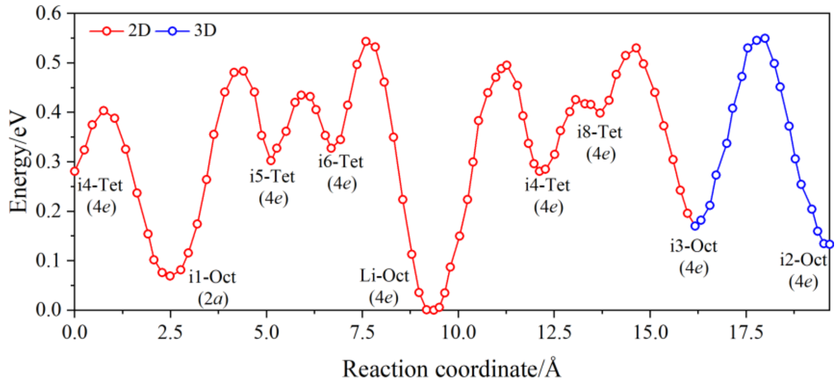

Figure S21. BVSE model of migration barriers for $LiAlBr_4$ using the models taken from the SPXD and PND data. The relative site energy is referred to zero for Li^+ lattice sites.

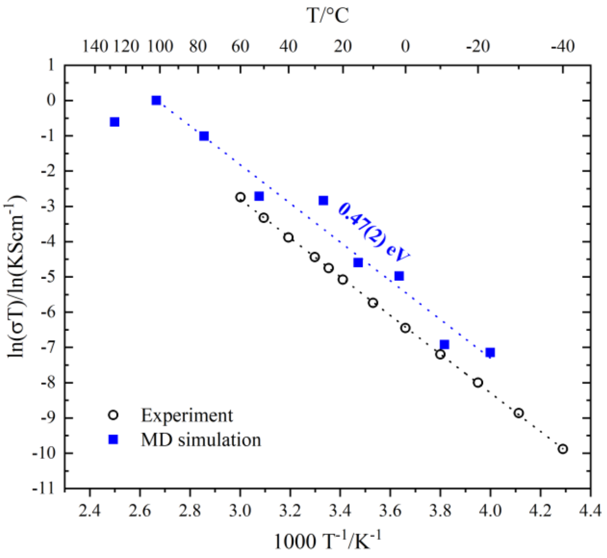

Figure S22. Comparison of Arrhenius plots of conductivity values obtained for $LiAlCl_4$ from temperature-dependent molecular dynamics simulations (filled squares) and impedance spectroscopy (open circles).

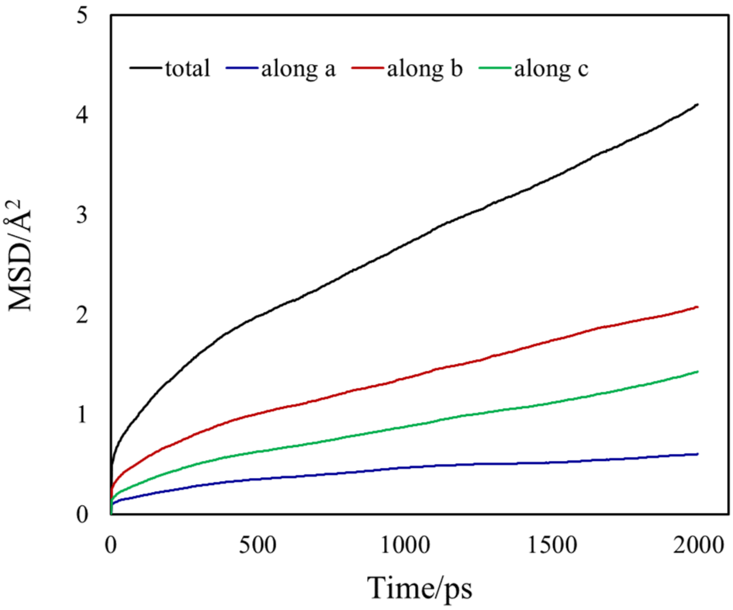

Figure S23. Anisotropy of the mean squared displacement (MSD) of Li^+ ions in $LiAlCl_4$ at 350 K.

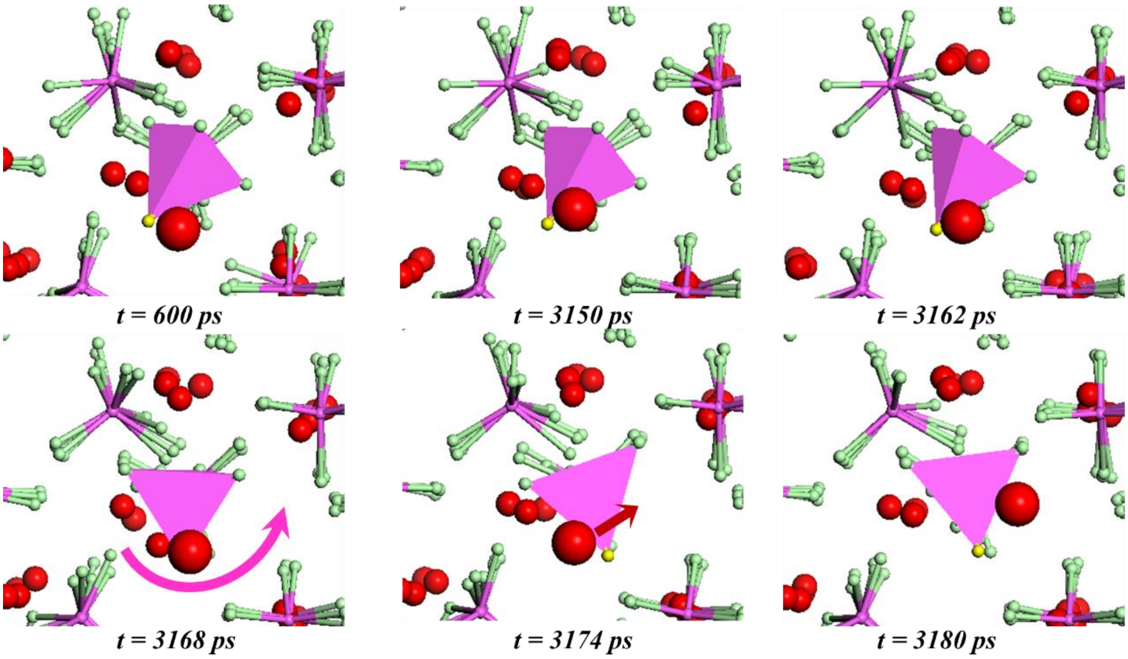

Figure S24. Details from MD simulations of $LiAlCl_4$ at T = 275 K exemplifying the correlation between $[AlCl_4]^-$ rotations and Li^+ hops.

Table S1. Experimental parameters for the synthesis of $LiAlX_4$ (X = Cl, Br, I).

Sample ID	Reagents	N° balls	Speed / rpm	Milling time / h	Ball:powder ratio
**1**	1:1 LiCl:$AlCl_3$	10	450	1	80:1
**2**	1:1 LiCl:$AlCl_3$	10	450	3	80:1
**3**	1:1 LiCl:$AlCl_3$	10	450	5	80:1
**4**	1:1 LiBr:$AlBr_3$	10	450	3	80:1
**5**	1:1 LiBr:$AlBr_3$	10	450	6	80:1
**6**	1:1 LiBr:$AlBr_3$	10	450	9	80:1
**7**	1:1 LiBr:$AlBr_3$	10	300	4	80:1
**8**	1:1 LiBr:$AlBr_3$	10	300	6	80:1
**9**	1:1 LiBr:$AlBr_3$	10	300	8	80:1
**10**	1:1 LiI:AlI_3	6	300	1.5	80:1
**11**	1:1 LiI:AlI_3	6	300	3	80:1
**12**	1:1 LiI:AlI_3	6	300	6	80:1
**13**	1:1 LiI:AlI_3	10	200	1.5	80:1
**14**	1:1 LiI:AlI_3	10	200	3	80:1
**15**	1:1 LiI:AlI_3	10	200	6	80:1

Table S2. Colours of as-synthesised $LiAlX_4$ (X = Cl, Br, I). samples used for characterisation and measurements.

Sample ID	Composition	Colour
**2**	$LiAlCl_4$	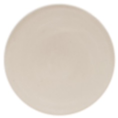
**8**	$LiAlBr_4$	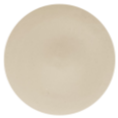
**15**	$LiAlI_4$	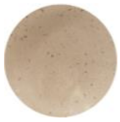

Table S3. Crystallographic data from Rietveld refinement of $LiAlCl_4$ against SPXD and PND data.

	**Literature**[1, 18†]	**SPXD**	**PND**
Chemical formula		$LiAlCl_4$	
Crystal system		Monoclinic	
Space group		$P2_1/c$ (14)	
Z		4	
a / Å	7.007(3)	7.0044(1)	7.0011(3)
b / Å	6.504(4)	6.5074(1)	6.5042(3)
c / Å	12.995(10)	12.995(1)	12.9884(6)
β / °	93.32(5)	93.315(1)	93.321(2)
V / Å^3	591	591.34(2)	590.45(6)
Calculated density, ρ / g cm^{-3}	1.98	1.9739	1.977
Observations	1434	10001	4177
Parameters	–	64	77
Phase fraction	–	0.99(1)	0.97(1)
R_{wp}	–	0.0228	0.0152(total)
χ^2	–	5.01	1.09

† Single crystal data.

Table S4. Atomic parameters of $LiAlCl_4$ obtained from Rietveld refinement against PND data.

**Atom**	**Wyckoff**	***x***	***y***	***z***	U_{iso} / Å^2 *
Cl1	4*e*	0.6967(6)	0.1802(6)	0.0458(3)	0.02587*
Cl2	4*e*	0.8074(8)	0.6315(5)	0.9302(4)	0.02571*
Cl3	4*e*	0.9214(6)	0.1816(7)	0.8106(3)	0.03049*
Cl4	4*e*	0.4387(7)	0.3138(6)	0.8107(3)	0.0233*
Al	4*e*	0.7074(7)	0.3234(1)	0.9011(4)	0.012(1)
Li	4*e*	0.158(2)	0.007(3)	0.383(2)	0.039(4)

* denotes that U_{iso} values were refined anisotropically.

Table S5. Refined anisotropic displacement parameters of $LiAlCl_4$ from PND data (U_{ij} / Å^2).

**Atom**	U_{11}	U_{22}	U_{33}	U_{12}	U_{13}	U_{23}
Cl1	0.018(2)	0.050(3)	0.010(2)	-0.008(2)	0.004(2)	0.013(2)
Cl2	0.027(2)	0.010(2)	0.039(2)	0.004(2)	-0.005(2)	0.010(2)
Cl3	0.011(2)	0.060(3)	0.022(2)	-0.002(2)	0.008(1)	-0.016(3)
Cl4	0.013(2)	0.032(3)	0.023(2)	0.010(2)	-0.014(1)	-0.006(2)

Table S6. Atomic parameters of $LiAlCl_4$ obtained from Rietveld refinement against SPXD data.

**Atom**	**Wyckoff**	***x***	***y***	***z***	**U_{iso} / Å^2**
Cl1	4*e*	0.6938(2)	0.1835(3)	0.0468(1)	0.044*
Cl2	4*e*	0.8084(2)	0.6311(3)	0.9271(1)	0.047*
Cl3	4*e*	0.9241(2)	0.1806(3)	0.8139(1)	0.043*
Cl4	4*e*	0.4390(2)	0.3163(3)	0.8128(1)	0.046*
Al	4*e*	0.7036(2)	0.3204(3)	0.8993(1)	0.0365(5)
Li	4*e*	0.158	0.007	0.383	0.039

* denotes that U_{iso} values were refined anisotropically.

Table S7. Refined anisotropic displacement parameters of $LiAlCl_4$ from SPXD data (U_{ij} / Å^2).

**Atom**	**U_{11}**	**U_{22}**	**U_{33}**	**U_{12}**	**U_{13}**	**U_{23}**
Cl1	0.047(2)	0.065(2)	0.021(2)	-0.016(2)	0.002(1)	0.002(1)
Cl2	0.041(2)	0.058(2)	0.044(2)	0.009(2)	-0.009(1)	0.005(2)
Cl3	0.028(1)	0.071(2)	0.031(1)	-0.003(2)	0.008(1)	-0.005(2)
Cl4	0.020(1)	0.067(2)	0.052(1)	0.005(2)	0.003(1)	-0.009(8)

Table S8. Comparison between bond lengths and angles from SPXD and PND data in $LiAlCl_4$.

**Bond**	**SPXD / Å**	**PND / Å**	**Angle**	**SPXD / °**	**PND / °**
**Al-Cl1**	2.119(2)	2.103(8)	**Cl1-Al-Cl2**	105.6(1)	106.5(3)
**Al-Cl2**	2.174(2)	2.149(9)	**Cl1-Al-Cl3**	110.48(9)	111.0(4)
**Al-Cl3**	2.154(2)	2.163(7)	**Cl1-Al-Cl4**	113.75(9)	113.5(3)
**Al-Cl4**	2.112(2)	2.159(6)	**Cl2-Al-Cl3**	103.5(1)	105.3(3)
**Li-Cl1**	2.500(1)	2.51(2)	**Cl2-Al-Cl4**	112.2(1)	112.8(4)
**Li-Cl2**	2.595(2)	2.56(2)	**Cl3-Al-Cl4**	110.7(1)	107.6(3)
**Li-Cl2**	2.701(2)	2.72(2)			
**Li-Cl3**	2.864(2)	2.82(2)			
**Li-Cl3**	2.730(2)	2.75(2)			
**Li-Cl4**	2.497(2)	2.51(2)			

Table S9. Crystallographic data from Rietveld refinement of $LiAlBr_4$ against SPXD and PND data

	**SPXD**	**PND**
Chemical formula	$LiABr_4$	
Crystal system	Monoclinic	
Space group	$P2_1/c$ (14)	
Z	4	
a/Å	7.4286(1)	7.4358(4)
b/Å	6.8866(1)	6.8930(4)
c/Å	13.7275(2)	13.7385(9)
β/°	93.2049(9)	93.212(3)
V/Å^3	701.17(2)	703.1(1)
Calculated density, ρ/gcm^{-3}	3.349	3.340
Observations	10001	5858
Parameters	64	76
Phase fraction	0.97(1)	0.97(1)
R_{wp}	0.0207	0.0176 (total)
χ^2	4.42	1.35

Table S10. Atomic parameters of $LiAlBr_4$ obtained from Rietveld refinement against PND data.

**Atom**	**Wyckoff**	***x***	***y***	***z***	U_{iso} / Å^2
Br1	4*e*	0.6980(4)	0.3354(6)	0.5476(3)	0.012(1)
Br2	4*e*	0.8084(7)	0.8547(8)	0.4365(4)	0.027(1)
Br3	4*e*	0.9356(8)	0.3355(7)	0.3135(4)	0.034(1)
Br4	4*e*	0.4374(8)	0.2125(5)	0.3089(3)	0.0122(9)
Al	4*e*	0.7062(6)	0.175(1)	0.4001(3)	0.013(1)
Li	4*e*	0.907(2)	0.975(3)	0.622(1)	0.040(3)

Table S11. Atomic parameters of $LiAlBr_4$ obtained from Rietveld refinement against SPXD data.

**Atom**	**Wyckoff**	***x***	***y***	***z***	U_{iso} / Å^2
Br1	4*e*	0.6987(3)	0.3218(2)	0.5507(2)	0.046*
Br2	4*e*	0.8060(3)	0.8635(2)	0.4292(2)	0.040*
Br3	4*e*	0.9337(3)	0.3204(3)	0.3140(2)	0.046*
Br4	4*e*	0.4410(3)	0.1890(3)	0.3115(2)	0.044*
Al	4*e*	0.7112(4)	0.1838(6)	0.3997(2)	0.040(1)
Li	4*e*	0.907	0.975	0.622	0.040

* denotes that U_{iso} values were refined anisotropically.

Table S12. Refined anisotropic displacement parameters of $LiAlBr_4$ from SPXD data (U_{ij} / Å^2).

Atom	U_{11}	U_{22}	U_{33}	U_{12}	U_{13}	U_{23}
Br1	0.054(2)	0.062(2)	0.021(2)	0.005(2)	-0.009(1)	-0.012(1)
Br2	0.033(2)	0.045(2)	0.042(2)	0.003(2)	-0.007(1)	0.008(2)
Br3	0.036(2)	0.062(2)	0.040(1)	-0.024(2)	0.009(1)	0.018(2)
Br4	0.016(1)	0.053(2)	0.062(2)	-0.005(1)	-0.002(1)	-0.016(2)

Table S13. Comparison between bond lengths and angles from SPXD and PND data in $LiAlBr_4$.

Bond	SPXD / Å	PND / Å	Angle	SPXD / °	PND / °
Al-Br1	2.287(4)	2.312(7)	Br1-Al-Br2	105.2(2)	106.5(3)
Al-Br2	2.344(4)	2.379(9)	Br1-Al-Br3	111.3(2)	105.6(3)
Al-Br3	2.285(4)	2.403(7)	Br1-Al-Br4	113.3(2)	110.9(3)
Al-Br4	2.286(4)	2.314(7)	Br2-Al-Br3	104.9(2)	107.7(3)
Li-Br1	2.981(2)	3.07(2)	Br2-Al-Br4	110.7(2)	118.3(3)
Li-Br2	2.539(2)	2.59(2)	Br3-Al-Br4	111.1(2)	107.2(3)
Li-Br2	2.817(2)	2.74(2)			
Li-Br3	2.489(2)	2.58(2)			
Li-Br3	2.986(2)	2.94(2)			
Li-Br4	3.009(2)	3.07(2)			

Table S14. Crystallographic data from Rietveld refinement of $LiAlI_4$ against SPXD and PND data.

	**SPXD**	**PND**
Chemical formula	$LiAlI_4$	
Crystal system	Monoclinic	
Space group	$P2_1/c$ (14)	
Z	4	
a/Å	8.0846(1)	8.0771(6)
b/Å	7.4369(1)	7.4314(6)
c/Å	14.8890(2)	14.882(1)
β/°	93.0457(8)	93.070(4)
V/Å^3	893.92(3)	892.0 (1)
Calculated density, ρ/gcm^{-3}	4.0238	4.033
Observations	13612	8770
Parameters	64	85
Phase fraction	1.00	0.99(1)
R_{wp}	0.03185	0.0162 (total)
χ^2	7.82	1.03

Table S15. Atomic parameters of $LiAlI_4$ obtained from Rietveld refinement against PND data.

**Atom**	**Wyckoff**	x	y	z	U_{iso} / Å^2
I1	4*e*	0.6966(6)	0.3365(8)	0.5508(3)	0.014(1)
I2	4*e*	0.8029(8)	0.843(1)	0.4369(4)	0.026(2)
I3	4*e*	0.9349(8)	0.3360(8)	0.3129(5)	0.028(2)
I4	4*e*	0.4379(7)	0.2080(6)	0.3089(4)	0.010(1)
Al	4*e*	0.7074(5)	0.173(1)	0.4020(3)	0.013(1)
Li	4*e*	0.869(3)	0.970(2)	0.616(2)	0.065(5)

Table S16. Atomic parameters of $LiAlI_4$ obtained from Rietveld refinement against SPXD data.

**Atom**	**Wyckoff**	x	y	z	U_{iso} / Å^2
I1	4*e*	0.6958(4)	0.3247(3)	0.5569(2)	0.044*
I2	4*e*	0.8050(3)	0.8539(3)	0.4297(2)	0.043*
I3	4*e*	0.9378(4)	0.3245(3)	0.3139(2)	0.047*
I4	4*e*	0.4352(3)	0.1882(3)	0.3091(2)	0.046*
Al	4*e*	0.7140(7)	0.1886(10)	0.3967(4)	0.035(2)
Li	4*e*	0.869	0.970	0.616	0.065

* denotes that U_{iso} values were refined anisotropically.

Table S17. Refined anisotropic displacement parameters of $LiAlI_4$ from SPXD data (U_{ij} / Å^2).

Atom	U_{11}	U_{22}	U_{33}	U_{12}	U_{13}	U_{23}
I1	0.055(2)	0.045(2)	0.033(2)	0.002(2)	0.004(2)	-0.008(2)
I2	0.015(1)	0.041(2)	0.072(3)	0.010(2)	-0.016(2)	-0.010(2)
I3	0.083(2)	0.042(2)	0.016(2)	-0.026(2)	-0.005(2)	0.016(2)
I4	0.007(1)	0.046(2)	0.086(2)	-0.003(2)	-0.003(1)	-0.018(2)

Table S18. Comparison between bond lengths and angles from SPXD and PND data in $LiAlI_4$.

Bond	SPXD / Å	PND / Å	Angle	SPXD / °	PND / °
Al-I1	2.601(7)	2.531(7)	I1-Al-I2	103.0(2)	107.4(3)
Al-I2	2.636(7)	2.62(1)	I1-Al-I3	112.2(3)	106.2(3)
Al-I3	2.460(7)	2.620(8)	I1-Al-I4	112.3(2)	111.2(3)
Al-I4	2.544(6)	2.531(7)	I2-Al-I3	106.0(2)	109.0(3)
Li-I1	3.086(2)	3.19(2)	I2-Al-I4	109.0(3)	116.2(3)
Li-I2	3.049(3)	3.13(2)	I3-Al-I4	113.5(3)	106.4(3)
Li-I2	2.927(3)	2.85(2)			
Li-I3	2.861(3)	2.94(2)			
Li-I3	3.336(3)	3.29(2)			
Li-I4	2.998(3)	3.07(2)			

Table S19. Bond-valence sums for $LiAlX_4$ (X = Cl, Br, I) obtained from Rietveld refinement against PND data.

Atom	$LiAlCl_4$	$LiAlBr_4$	$LiAlI_4$
X1	-0.99	-0.88	-0.92
X2	-1.06	-1.09	-1.06
X3	-0.94	-0.98	-0.95
X4	-0.93	-0.87	-0.93
Al	3.00	2.84	2.91
Li	0.93	0.98	0.96

Table S20. Atomic coordinates and relative energies of the available interstitial sites in $LiAlCl_4$, as estimated from SoftBV-GUI.

Label	Wyckoff	*x*	*y*	*z*	Relative site energy / eV
i1	2*a*	0	0.5	0.5	0.110
i2	4*e*	0.236	0.014	0.792	0.161
i3	4*e*	0.208	0.333	0.424	0.200
i4	4*e*	0.597	0.903	0.868	0.214
i5	4*e*	0.028	0.847	0.847	0.225
i6	4*e*	0.847	0.833	0.785	0.261
i7	4*e*	0.639	0.347	0.194	0.391
i8	4*e*	0.472	0.833	0.333	0.430
i9	4*e*	0.500	0.319	0.160	0.430
i10	4*e*	0.556	0.514	0.028	0.968
i11	2*d*	0.5	0.5	0	0.982

Table S21. Atomic coordinates and relative energies of the available interstitial sites in $LiAlBr_4$, as estimated from SoftBV-GUI.

Label	Wyckoff	*x*	*y*	*z*	Relative site energy / eV
i1	2*a*	0	0	0	0.077
i2	4*e*	0.240	0.000	0.785	0.143
i3	4*e*	0.604	0.931	0.868	0.179
i4	4*e*	0.219	0.167	0.924	0.268
i5	4*e*	0.021	0.833	0.840	0.294
i6	4*e*	0.844	0.847	0.785	0.314
i7	4*e*	0.344	0.639	0.806	0.379
i8	4*e*	0.375	0.167	0.972	0.414
i9	4*e*	0.510	0.333	0.160	0.425
i10	2*d*	0.5	0.5	0	0.619

Table S22. Atomic coordinates and relative energies of the available interstitial sites in $LiAlI_4$, as estimated from SoftBV-GUI.

**Label**	**Wyckoff**	***x***	***y***	***z***	**Relative site energy / eV**
i1	2*a*	0	0.5	0.5	0.044
i2	4*e*	0.240	0.000	0.774	0.120
i3	4*e*	0.604	0.948	0.869	0.160
i4	2*d*	0.5	0.5	0	0.320
i5	4*e*	0.219	0.167	0.923	0.336
i6	4*e*	0.979	0.167	0.161	0.359
i7	4*e*	0.854	0.844	0.786	0.393
i8	4*e*	0.344	0.854	0.298	0.408
i9	4*e*	0.385	0.167	0.970	0.409
i10	4*e*	0.500	0.844	0.339	0.444

Table S23. Forcefield employed in the Molecular dynamics simulations conducted using the GULP simulation software.

```
# softBV forcefield generated for MD using GULP
# Turn off Ewald sum and charged interactions

keyword no electrostatics species
Al core  1.59245043
Cl core -0.62796303
Li core  0.91940169

Morse force
Al core Cl core  1.494398  1.547988  2.010575  0.00000  0.00000  11.000000
Li core Cl core  0.479124  1.577287  2.465906  0.00000  0.00000  11.000000

eam_density exponential 0
Al core  Cl core  1  1.547988  1.896060
Li core  Cl core  1  1.577287  1.648290

eam_functional banerjea_smith 0.5
Al core  86.0773  0  3
Li core  40.4764  0  1
Cl core  59.0263  0  1

qerfc force
# fractional radius = 0.795
Al core Al core  1.7649  11.000000
Al core Li core  1.9239  11.000000
Cl core Cl core  2.7666  11.000000
Li core Li core  2.0829  11.000000

three cosine
Al core Cl core Cl core  79.00  109.500  0.000  2.900  0.000  2.90  0.000  3.8  0  0
```

References

1. Mairesse, G.; Barbier, P.; Vignacourt, J. P.; Baert, F., Lithium Tetrachloroaluminate, $LiAlCl_4$. *Cryst. Struct. Commun.* **1977,** *6*, 15.

2. Scholz, F.; Unkrig, W.; Eiden, P.; Schmidt, M. A.; Garsuch, A.; Krossing, I., Synthesis, Spectroscopic Characterization, Crystal Structures, Energetics, and Thermal Stabilities of Li[AlX_4] (X = Cl, Br): Investigation and Performance of Their Electrolyte Solutions. *Eur. J. Inorg. Chem.* **2015,** *2015*, 3128-3138.

3. Begun, G. M.; Boston, C. R.; Torsi, G.; Mamantov, G., Raman spectra of molten aluminum trihalide-alkali halide systems. *Inorg. Chem.* **1971,** *10*, 886-889.

4. Suryanarayana, C., Mechanical alloying and milling. *Prog. Mater Sci.* **2001,** *46*, 1-184.

5. Boef, G.; Bruins Slot, H.; Doctors van Leeuwen, R. A. W.; Wessels, H.; van Spronsen, J. W., Thermische Untersuchung einiger Systeme. I. Alkalihalogenid-Aluminiumhalogenid. *Z. Anorg. Allg. Chem.* **1967,** *353*, 93-102.

6. Katsenis, A. D.; Puškarić, A.; Štrukil, V.; Mottillo, C.; Julien, P. A.; Užarević, K.; Pham, M.-H.; Do, T.-O.; Kimber, S. A. J.; Lazić, P.; Magdysyuk, O.; Dinnebier, R. E.; Halasz, I.; Friščić, T., In situ X-ray diffraction monitoring of a mechanochemical reaction reveals a unique topology metal-organic framework. *Nat. Commun.* **2015,** *6*, 6662.

7. Willmott, P. R.; Meister, D.; Leake, S. J.; Lange, M.; Bergamaschi, A.; Boge, M.; Calvi, M.; Cancellieri, C.; Casati, N.; Cervellino, A.; Chen, Q.; David, C.; Flechsig, U.; Gozzo, F.; Henrich, B.; Jaggi-Spielmann, S.; Jakob, B.; Kalichava, I.; Karvinen, P.; Krempasky, J.; Ludeke, A.; Luscher, R.; Maag, S.; Quitmann, C.; Reinle-Schmitt, M. L.; Schmidt, T.; Schmitt, B.; Streun, A.; Vartiainen, I.; Vitins, M.; Wang, X.; Wullschleger, R., The Materials Science beamline upgrade at the Swiss Light Source. *J. Synchrotron Rad.* **2013,** *20*, 667-682.

8. Smith, R. I.; Hull, S.; Tucker, M. G.; Playford, H. Y.; McPhail, D. J.; Waller, S. P.; Norberg, S. T., The upgraded Polaris powder diffractometer at the ISIS neutron source. *Rev. Sci. Instrum.* **2019,** *90*, 115101.

9. Arnold, O.; Bilheux, J. C.; Borreguero, J. M.; Buts, A.; Campbell, S. I.; Chapon, L.; Doucet, M.; Draper, N.; Ferraz Leal, R.; Gigg, M. A.; Lynch, V. E.; Markvardsen, A.; Mikkelson, D. J.; Mikkelson, R. L.; Miller, R.; Palmen, K.; Parker, P.; Passos, G.; Perring, T. G.; Peterson, P. F.; Ren, S.; Reuter, M. A.; Savici, A. T.; Taylor, J. W.; Taylor, R. J.; Tolchenov, R.; Zhou, W.; Zikovsky, J., Mantid—Data analysis and visualization package for neutron scattering and μSR experiments. *Nucl. Instrum. Meth. A.*, **2014,** *764*, 156-166.

10. Rietveld, H., A profile refinement method for nuclear and magnetic structures. *J. Appl. Crystallogr.* **1969,** *2*, 65-71.

11. Larson, A. C.; von Dreele, R. B., *General Structure Analysis System (GSAS), Los Alamos National Laboratory Report LAUR*. Los Alamos National Laboratory Report LAUR: Los Alamos, NM, USA, 1995.

12. Toby, B., EXPGUI, a graphical user interface for GSAS. *J. Appl. Crystallogr.* **2001,** *34*, 210-213.

13. Toby, B. H.; Von Dreele, R. B., GSAS-II: the genesis of a modern open-source all purpose crystallography software package. *J. Appl. Crystallogr.* **2013,** *46*, 544-549.

14. McCusker, L. B.; Von Dreele, R. B.; Cox, D. E.; Louer, D.; Scardi, P., Rietveld refinement guidelines. *J. Appl. Crystallogr.* **1999,** *32*, 36-50.

15. Tanibata, N.; Takimoto, S.; Nakano, K.; Takeda, H.; Nakayama, M.; Sumi, H., Metastable Chloride Solid Electrolyte with High Formability for Rechargeable All-Solid-State Lithium Metal Batteries. *ACS Materials Lett.*, **2020**, 880-886.

16. Gale, J. D.; Rohl, A. L., The General Utility Lattice Program (GULP). *Mol. Simul.* **2003,** *29*, 291-341.

17. Flores-González, N., Insights into the structure of mechanochemically-synthesised $LiAlX_4$ (X = Cl, Br, I) by powder neutron diffraction. STFC ISIS Neutron and Muon Source, https://doi.org/10.5286/ISIS.E.RB1890320-1 (X = Cl), https://doi.org/10.5286/ISIS.E.RB1890321-1 (X = Br), https://doi.org/10.5286/ISIS.E.RB1890322-1 (X = I): 2018.

18. Mairesse, G.; Barbier, P.; Wignacourt, J.-P., Comparison of the crystal structures of alkaline (M = Li,Na,K,Rb,Cs) and pseudo-alkaline (M = NO, NH_4) tetrachloroaluminates, $MAlCl_4$. *Acta Crystallogr., Sect. B: Struct. Sci., Cryst. Eng. Mater.* **1979,** *35*, 1573-1580.
